# Active Play in a Digital Age, Exploring Children’s (Aged 8-13 Years) Views of a Physical Activity App: Qualitative Formative Study

**DOI:** 10.2196/76498

**Published:** 2025-11-11

**Authors:** Marie T Frazer, Lauren Charlesworth, Luca Wilson, Jennifer Hall, Farwa Batool, Mikel Subiza-Perez, Jan Burkhardt, Andy Daly-Smith, Anna Chalkley

**Affiliations:** 1 Faculty of Health Studies, Institute for Health and Social Care, University of Bradford, Richmond Road, Bradford, BD7 1DP, United Kingdom, 44 1274 232323; 2 Bradford Centre for Qualitative Research, Wolfson Centre for Applied Health Research, Bradford, United Kingdom; 3 Bradford Institute for Health Research, Bradford Teaching Hospitals NHS Foundation Trust, Bradford, United Kingdom; 4 Department of Clinical and Health Psychology and Research Methods, University of the Basque Country UPV/EHU, Donostia-San Sebastián, Spain; 5 Spanish Consortium for Research on Epidemiology and Public Health, Instituto de Salud Carlos III, Madrid, Spain; 6 Biogipuzkoa Health Research Institute, Group of Public Health and Environmental Epidemiology, Donostia- San Sebastián, Spain; 7 Centre for Applied Education Research, Wolfson Centre for Applied Health Research, Bradford, United Kingdom

**Keywords:** children, young people, physical activity, mHealth, citizen science, feasibility, acceptability, usability, behaviour change, mobile health

## Abstract

**Background:**

The use of smartphones and interest in mobile health (mHealth) has grown in recent years with physical activity apps demonstrating potential to facilitate behavior change. However, there remains limited understanding of what specifically motivates children to engage meaningfully with these tools.

**Objective:**

This qualitative formative study aimed to determine children’s perceptions of a bespoke physical activity mHealth app (Bestlife; Dubbit). It sought to explore the app’s appeal, functionality, and potential to support behavior change among children aged 8‐13 years.

**Methods:**

A total of 68 Young Citizen Scientists (YCSs) aged 8‐13 years were recruited from 5 schools (3 primary and 2 secondary) in Bradford, United Kingdom, through purposive sampling as part of a whole-system physical activity program (Join Us: Move. Play; JU:MP). Recruitment procedures were school-led, incorporating consented whole-class involvement at primary level and teacher-nominated groups at secondary level. YCSs were asked to download and explore the Bestlife app 1‐2 weeks before the school-based research session, completing a booklet to capture their experiences and those of their families. A total of 13 focus groups were conducted across 5 schools to explore children’s views in depth. The focus groups were designed to investigate children’s perceptions of the app. Qualitative data were analyzed inductively and deductively: An initial inductive analysis identified emerging themes, which were then mapped onto a framework of feasibility, usability, acceptability, and behavior change.

**Results:**

A total of 68 children (60 from primary schools and 8 from secondary schools) participated in the study. The study identified key factors influencing the feasibility, acceptability, usability, and behavior change potential of the Bestlife app among children. Feasibility was hindered by the parental email requirement during registration, which limited autonomy for older children. Acceptability was driven by gamified features, proportional rewards, and avatar customization, though participants requested more personalization to promote cultural inclusion and dynamic updates, linked to seasonal themes. Usability findings showed the interface was intuitive, with features promoting social interaction and competition enhancing engagement. However, younger users experienced navigational challenges, underscoring the need for clearer guidance. The app effectively incorporated behavior change techniques, including goal-setting, self-monitoring, and social collaboration, but required adjustments, such as reducing the frequency of emotional tracking prompts.

**Conclusions:**

The Bestlife app shows potential as an mHealth intervention for promoting physical activity in children. Enhancing cultural representation, simplifying onboarding processes, and refining engagement strategies could strengthen both uptake and sustained use. These findings highlight the importance of integrating user feedback into the iterative design process to optimize digital health tools for young populations. Further longitudinal research is recommended to evaluate longer-term engagement with the app, its impact on physical activity levels, and behavior change sustainability.

## Introduction

### Background

Physical inactivity may have profound consequences for children’s health, including reduced cardiometabolic fitness, impaired bone health, and increased adiposity [1,2]. It also poses risks to mental health, potentially leading to cognitive deficits and other negative psychological outcomes. These issues are particularly acute in areas with high deprivation and among ethnic minority communities, where inactivity levels are highest [3,4]. Conversely, regular physical activity during childhood provides lasting benefits, often persisting into adulthood and reducing the risk of noncommunicable diseases [5-7].

Across England, 53% of children aged 5‐16 years do not achieve the recommended 60 minutes of daily physical activity [8]. Social, economic, and cultural disparities significantly influence physical activity levels, with children living in areas of high socioeconomic deprivation and diverse ethnic minority communities, particularly those of South Asian heritage, experiencing lower levels of physical activity [8,9]. These disparities exacerbate health inequalities by limiting access to resources and culturally-tailored opportunities for physical activity, underscoring the urgent need for inclusive and targeted interventions [8,9].

To address the low levels of inactivity, the World Health Organization’s (WHO) Global Action Plan on Physical Activity 2018‐2030 advocates for the adoption of a whole systems approach to tackling physical inactivity, including incorporating the use of digital innovations to promote and support people of all ages to be more active and to build upon the rapidly growing practice of mHealth (mobile health) [10,11]

Smartphones are ubiquitous in young people’s lives, with 98% of 12‐15-year-olds having access to smartphones [12]. These devices provide quick access to information and social connections [13-15]. The dichotomous influence of smartphones on young people’s physical activity behavior is striking. For example, they are associated with increased screen time and decreased physical activity, as the ability to engage with peers and content online reduces opportunities for movement [14,16]. While digital tools can support healthier behaviors, it is vital to address the ethical considerations surrounding children’s use of smartphones. The increasing prevalence of smartphone use among individuals 16 years and younger has been linked to adverse effects on mental health, sleep quality, and addiction-like behaviors [17-20]. Given these risks, Bestlife (Dubbit) is not intended to promote unsupervised smartphone use. Rather, it is designed as a supportive tool to be used under parental or educator guidance, aligning with evidence that highlights the importance of adult mediation in reducing harm and fostering healthy digital habits [21]. However, smartphones also hold immense untapped potential as tools for positive health behavior change, including increasing physical activity [16,17]. When designed effectively, mHealth interventions can transform smartphones into facilitators of physical activity, using their accessibility, interactivity, and personalized features to promote engagement and behavior change [15,16].

Children from lower socioeconomic groups are particularly likely to use smartphones and tend to use them more frequently than their peers from other socioeconomic backgrounds [22]. Sharma et al [23] reiterated the increasing use of smart devices to access the internet among diverse low-income communities. This presents a possible opportunity to harness smartphones to promote healthy behavior, specifically within this demographic, and thus reduce health inequalities. Advances in technology and the widespread increase in smartphone ownership among young people [24] now enable the development of tailored interventions that address health disparities. By leveraging smartphones as accessible, interactive, and engaging tools, under parental supervision, it is possible to encourage positive behavior changes, particularly in physical activity, among younger individuals from low socioeconomic backgrounds.

Comprehensive reviews of physical activity apps suggest that many are commercially developed without academic input [25,26]. It has also been noted that many app developments do not make use of relevant underlying theoretical models to inform design elements used in apps [27]. Historically, many of these apps lack a solid foundation in behavioral science or public health principles, which raises concerns about effectiveness and appropriateness as tools for behavior change [28,29]. Recent trends, however, suggest a promising shift toward incorporating user-centered design and evidence-based theories. These approaches aim to enhance app functionality and engagement, particularly for children and adolescents, where the application of behavior change techniques remains in its infancy [30]. For example, the Snacktivity project is a notable case where researchers and health professionals collaborated to create an app promoting small, frequent bouts of activity “snacks” [31]. Other mHealth interventions, such as the Active 10 app developed by Public Health England, also demonstrate practitioner involvement [32].

Evaluations of physical activity apps are crucial to determine their effectiveness in promoting physical activity among users, with augmented reality games like Pokémon GO (Niantic) demonstrating a general tendency to positively affect physical activity levels among children and adolescents [33]. Many commercially available apps lack rigorous evidence of their efficacy, which raises concerns about their potential impact on children’s health behaviors. For instance, Schoffman et al [34] and Schoeppe et al [35] noted that despite the ubiquity of these apps, there is a significant gap in evidence demonstrating their effectiveness in real-world settings. Without proper evaluation, there is a risk that investment is made into interventions that children do not engage with and therefore do not effectively promote physical activity.

User engagement is a critical factor in the success of physical activity apps, especially for children who may have shorter attention spans and varying levels of motivation. Augmented reality displays and gamification features, as adjuncts, play a significant role in fostering user engagement with physical activity apps [36,37]. Both technologies leverage interactive elements and motivational design principles to make physical activity more enjoyable and encourage sustained participation [36,37]. Evaluating these apps can provide insights into usability and engagement factors that resonate with young users. Tong et al [38] found that apps with social networking features and personalized feedback significantly enhance user engagement. Understanding these dynamics is crucial for designing apps that children will want to use regularly.

Children have unique developmental needs and preferences that differ from those of adults. Evaluating physical activity apps aimed at children is necessary to ensure that the content is age-appropriate, engaging, and effective in motivating young users [39-41]. Many popular physical activity apps do not specifically target children, which indicates a lack of tailored interventions for this demographic [39]. This gap highlights the need for evaluations that focus on how well these apps meet the developmental and motivational needs of children. Furthermore, to ensure effective and equitable digital physical activity interventions for children, it is critical to address multifaceted barriers including socioeconomic disparities that disproportionately affect low socioeconomic status populations [42]. In addition, challenges related to eHealth literacy, language, and cultural representation significantly hinder engagement for Culturally and linguistically diverse communities [43], necessitating that future development prioritizes person-centered and participatory approaches involving target communities in the design process.

The Bestlife app was developed as a targeted response to the challenges young people face in engaging in physical activity. Designed for children aged 10‐13 years to use with parental guidance and delivered as part of the Join Us: Move. Play (JU:MP) whole system approach to improving children’s physical activity in Bradford, United Kingdom [44]. The app integrates physical activity tracking, gamified challenges, and emotional well-being assessments. Grounded in behavior change theory, the app incorporates evidence-based behavior change techniques [45] and was co-produced with children to ensure cultural relevance and user-centered design. This study adopted a citizen science approach to explore the app’s acceptability, feasibility, usability, and potential to facilitate behavior change among children, focusing particularly on their engagement with both physical activity and emotional well-being features.

### Aim

The primary aim of this study was to carry out a citizen science feasibility study of a children’s physical activity app, focusing on the perspectives of the users (ie, children). Specifically, we aimed to:

1. Assess the acceptability of the app, exploring children’s attitudes, preferences, and overall satisfaction, including how these factors influence their perceived engagement with physical activity and subjective well-being.
2. Evaluate the feasibility of incorporating the use of the app into children’s daily routines, and identify barriers and facilitators of use related to socioeconomic, cultural, and technological contexts.
3. Examine the app’s usability, focusing on navigation and ease of use.
4. Investigate early indicators of behavior change facilitated by the app, assessing user engagement with physical activity features.

## Methods

### The Bestlife App

Released in July 2023 on the Apple and Google Play Stores, the Bestlife app was developed by the software company Dubit in collaboration with the Born in Bradford (BiB) research program [46] and JU:MP. JU:MP [44] is a whole-system approach to increasing physical activity among children in Bradford, funded by Sport England as part of a local delivery pilot (LDP). The app was co-designed with researchers, practitioners, and, most importantly, children, ensuring its development was shaped by those who would use it.

Designed specifically for 10‐ to 13-year-olds, Bestlife integrates gamification, personalization, and emotional well-being tracking to encourage physical activity. The app enables users to engage in “active quests,” such as step challenges, fitness exercises, or visits to parks with QR codes, to earn points that can be used to customize their avatars. Parental permission, verified through email, is required to create an account.

Beyond solo challenges, Bestlife supports collaborative gameplay, fostering social engagement. Users track their activities and emotional well-being through an interactive dashboard, providing insights into their physical and mental health. The app aligns with JU:MP’s mission of improving children’s activity levels within their local environments, including schools, parks, and neighborhoods.

By integrating rewards, personalization, and emotional well-being tracking, Bestlife aims to increase children’s engagement in physical activity while fostering awareness of how participation relates to their mental health. For detailed screenshots and a guide on how to play (see Multimedia Appendix 1).

### Key Concepts

#### Acceptability, Feasibility, and Usability

Within this study, we used the key concepts of acceptability, feasibility, and usability as follows: Acceptability refers to how children react to the app, did they think it was appropriate, satisfactory, and attractive [47], including children’s willingness to download and engage with the app and their interest in participating in challenges or quests set on the app. Feasibility assesses the practicality of implementing the app within the target population’s daily routines and local context [47]. This includes smartphone ownership, internet accessibility, etc. Finally, usability focuses on the design aspects of the app that contribute to a positive user experience [48]. This consists of the ease of navigation, intuitiveness of the interface, and overall user-friendliness of the app. By examining these 3 dimensions, we aimed to provide a comprehensive assessment of the app’s potential for promoting physical activity among children and identify areas for improvement in future iterations.

#### Behavior Change Theory in the Development of Bestlife

The development of Bestlife was underpinned by the behavior change theory, specifically drawing on the Behavior Change Wheel (BCW) [45]. The BCW provides a systematic framework for designing interventions that support and sustain behavior change by addressing 3 core components: capability, opportunity, and motivation. These components form the COM-B (Capability, Opportunity, Motivation—Behavior) model, which posits that for a behavior to occur, an individual must have the capability (both physical and psychological), the opportunity (both social and environmental), and the motivation (both automatic and reflective) to engage in it. The Bestlife app was designed to integrate these elements, ensuring that it effectively encouraged young people to engage in and sustain physical activity.

Gamification elements such as points, rewards, and avatar customization were included to enhance motivation, providing immediate and tangible reinforcement for physical activity. The app also allowed users to track their own activity and emotional well-being, supporting self-monitoring and reflection, techniques recognized for fostering sustained behavior change. This design was informed by insights from Dubbit during the development phase, where children and young people recognized that being active made them feel happier, highlighting the potential for the app to reinforce that positive association and further motivate ongoing participation.

The design also considered the role of social and environmental opportunity in shaping behavior. By incorporating features that encouraged children to engage in activity within their local environment, such as interactive park visits using QR codes, the app aligned with JU:MP’s emphasis on creating active environments that make movement an embedded part of daily life. Ensuring accessibility was also a key principle, with the app designed to be used on smartphones, a device that is widely available to young people.

This approach ensured that the app aligned with the broader goals of JU:MP, leveraging a theory-driven yet practical design to maximize impact.

### Study Design

This study used a contributory citizen science approach, engaging children as citizen scientists. Citizen science, defined in this paper as collaboration between the public and professional scientists to advance research [49-51], has shown significant promise in engaging young people across various fields [52]. By capturing authentic insights from young people’s lived experiences, this method aligns with the growing recognition that young people should actively participate in research that impacts them [53]. Previous research has successfully applied citizen science to understand young people’s physical activity experiences [54-57]. This precedent suggests that a similar approach could be highly effective in evaluating the Bestlife app, providing valuable insights into its usability, effectiveness, and impact on physical activity levels among children and young people. The children involved in the research will be referred to as Young Citizen Scientists (YCSs) for the remainder of the paper.

### The Citizen Science Approach

#### Patient and Public Involvement (PPI)

A total of 3 discussion and consultation sessions were conducted before designing the study, with the aim of identifying barriers and facilitators to research participation with children in Bradford. These sessions, held between May and June 2023, engaged key stakeholders, including BiB Young Ambassadors, young people involved in a JU:MP longitudinal research study, and (primary and secondary) teachers. Insights from these sessions played a crucial role in shaping the study design to ensure the research process was engaging, relevant, and minimally disruptive.

The sessions with the BiB Young Ambassadors and the young people involved in JU:MP emphasized the importance of conducting the research in familiar settings, such as schools or community spaces, with adults the children already worked with. This approach aimed to create a comfortable and natural environment where children could speak freely. They also advised that all research materials, including consent forms, should be fun, engaging, and age-appropriate to maintain interest and understanding.

Teachers provided different suggestions based on the age group they worked with. Primary school teachers recommended conducting the sessions with the whole class to prevent lesson disruptions and to ensure that all children could take part. In contrast, secondary school teachers were keen to avoid taking an entire class out of a lesson, as this would put students out of sync with their peers. Instead, they suggested removing a small group of students for the session, allowing them to catch up on their work later with minimal disruption to the rest of the year group.

These insights informed the decision to use focus groups as the primary method of data collection, providing a comfortable and interactive format for children to share their perspectives. The use of booklets allowed information to be conveyed in an age-appropriate and engaging format. The booklets were colorful, featuring definitions of key terms such as “citizen science” and “JUMP,” as well as spaces for children to complete quizzes and record their own research, for example, whether members of their families enjoyed using the app (see Multimedia Appendix 2). This supported both understanding and active participation in the research. By incorporating these recommendations, the study design ensured that research activities were integrated smoothly into school routines while prioritizing a child-friendly and inclusive approach.

### Participants and Recruitment

The study targeted children and young people aged 8‐14 years (Key Stages 2 and 3) to become YCSs. Although the app was initially designed for children aged 10‐14 years, feedback from the public involvement groups suggested that younger children might also engage with the app, prompting the inclusion of 8 to 9-year-olds.

The sampling strategy was designed to ensure a diverse and representative sample of children from Bradford, aligning with the aims of the JU:MP whole-systems approach to increasing physical activity for all children and young people and particularly those from low socioeconomic status and ethnic minority groups. Given that Bestlife was being implemented within JU:MP delivery areas, recruitment was purposive, targeting schools already engaged with the intervention. This approach maximized feasibility by leveraging existing networks and relationships with schools, ensuring that the study was embedded within communities where the intervention was being delivered.

For primary schools, a 2-stage recruitment process was used. First, all schools JU:MP were working with at the time were invited to participate, with recruitment being dependent on the schools that responded first. Second, to ensure ethnic diversity in the sample, reflecting the demographics of Bradford, schools with different ethnic compositions were specifically sought, given JU:MP’s implementation teams’ knowledge of the schools and the catchment areas. Initial primary focus groups took place in a predominantly White British area, and to ensure representation from Bradford’s large South Asian population, an additional primary school was selected from areas with a higher proportion of Asian pupils. While the recruitment process was shaped by practical constraints, including school engagement and availability, the sampling strategy was purposeful in achieving variation across ethnic backgrounds. This ensured that the study captured a broad range of experiences and perspectives, making the findings more relevant for diverse communities.

A total of 2 secondary schools were contacted directly through existing links within JU:MP areas. Within the secondary schools, teachers facilitated recruitment. In one school, this was done by identifying a range of students from different socioeconomic and ethnic backgrounds from across year 8 (approximately two per form) and handing out the research booklets. In the second school, this involved an established lunchtime youth group, who were also given the research booklets. Focus groups were conducted in school-provided spaces with small groups of YCSs. In primary schools, entire classes participated in engaging with the app, with only consenting students included in the formal research.

### Ethical Considerations

Ethical considerations are fundamental, particularly when working with potentially vulnerable demographics such as children, as they protect participants’ rights, dignity, and well-being.

#### Ethics Review and Approvals

Ethical approval for all studies was granted by the University of Bradford Research Ethics Committee (Humanities, Social, and Health Sciences Research Ethics Panel; reference: E891; July 2021). Subsequent amendments, including the participant experience focus groups following the control trial and the JU:MP focus groups, were approved on June 29, 2022. Similarly, the Bestlife study amendment to E891 was approved on July 04, 2023.

#### Privacy and Confidentiality Protection

All data were anonymized, and participants’ identities were protected throughout the research process. Focus group discussions were audio-recorded and transcribed, and transcripts were anonymized before analysis. Participant booklets were collated and digitized, and both booklets and transcripts were coded to ensure the integration of verbal and written responses. Safeguarding procedures in line with university policy were in place throughout the research and would have been followed had any concerns arisen.

#### Compensation

Participants did not receive compensation for their involvement. This decision aligns with ethical principles to avoid undue influence on participation decisions, particularly as focus groups were conducted during the regular school day as part of existing school activities.

#### Identification in Images

No identification of individual participants or users is possible in any images within this manuscript. All images presented are illustrative of the research tools or conceptual frameworks and do not contain identifiable individuals.

### Study Procedure

The study followed a 2-phase data collection process, involving independent app use followed by facilitated focus groups.

#### Pre–Focus Group Engagement

Before the focus groups, YCSs were provided with a booklet containing an information sheet, consent form, and QR code to download Bestlife. The booklet guided them through independent app exploration for a week, prompting them to record their experiences, preferences, and interactions. It also encouraged them to discuss the app with their families and friends, capturing broader perceptions of its usability and appeal.

#### Focus Group Procedures

After the independent use phase, researchers conducted focus groups in schools to facilitate discussion and further app engagement.

- Primary school focus groups were conducted at the class level to ensure all children could participate without disrupting lesson timetables.
- Secondary school focus groups were held with smaller groups, as preferred by teachers to minimize classroom disruption.

Focus groups were selected based on public involvement (PPI) sessions, ensuring that children participated in a setting where they felt comfortable sharing their experiences. This approach was chosen over interviews to create a more natural and engaging environment for discussion [58]. A copy of the focus group guide can be found in Multimedia Appendix 3.

Focus groups were primarily led by MTF, a female PhD student with an MA in Education and extensive training in qualitative research. When multiple groups ran concurrently, MTF facilitated one, supported by 2 other trained female PhD students and research assistants. All facilitators had previous qualitative research experience and received specific training on focus group delivery and study objectives. No previous relationship existed between researchers and participants. Children were informed at the outset that the research aimed to explore their experiences with the Bestlife app. MTF, embedded within the JU:MP program, conducted this study as part of its broader evaluation.

During the sessions, researchers used the booklets as discussion prompts, encouraging children to expand on their experiences. The booklets also allowed children to express themselves nonverbally, either through writing or drawing, helping to facilitate engagement and accommodate different communication preferences [59]. In addition, focus groups incorporated live app demonstrations, where children interacted with Bestlife in real time, exploring its features and providing immediate feedback. This hands-on element enabled researchers to observe and record engagement, usability, and barriers to interaction through the use of reflective field notes.

In all focus groups, school staff such as support staff or class teachers were present to provide a familiar environment for children. However, school staff did not participate in the research discussions to ensure that children felt free to share their honest opinions. Each focus group session lasted approximately 30 minutes to 1 hour, depending on school scheduling, and was designed to fit within the standard school timetable.

By integrating both individual reflection and interactive group discussion, this procedure ensured a comprehensive evaluation of the Bestlife app, capturing insights into both independent use and collaborative engagement. After each focus group, members of the research team engaged in informal reflective discussions to consider group dynamics, emerging insights, and the facilitation process. Field notes were taken during and after the sessions to document observations, including participant engagement, nonverbal cues, and contextual details. These notes informed the interpretation of data and supported ongoing reflexivity throughout the study.

### Data Analysis

All focus group discussions were audio-recorded and transcribed. Transcripts were anonymized before analysis, and participant booklets were collated and digitized. Both the booklets and the transcripts were coded to ensure the integration of both verbal and written responses.

The analysis was conducted in 2 key phases. Initially, open coding was carried out by 2 researchers (MTF and LC) to capture emerging patterns in participant responses without predefined categories. This inductive process ensured that all relevant insights were captured, allowing themes to come directly from the data, prioritizing the children’s voices. Following the open coding, the generated codes were compared against the original theoretical framework underpinning the Bestlife app, which was structured around feasibility, acceptability, usability, and behavior change (done by MTF, LW, and AC). The initial open codes aligned well with these categories.

In the second phase, researchers (LW and MTF) recoded the data, explicitly mapping it onto these 4 overarching categories. The recoding process ensured that all identified insights were systematically integrated into the study’s core evaluative framework. This theory-driven recoding ensured initial themes were aligned with the study’s predefined evaluation framework, while the first phase allowed the researchers to be open to organic themes that the children had discussed. A summary of example codes is available in the code book (see Multimedia Appendix 4).

## Results

### Overview

A total of 68 YCSs gave consent to take part in the research. In total, 60 were from 2 primary schools and 8 were from 2 secondary schools (see Table 1)

**Table 1.** Number of focus groups and Young Citizen Scientists by school and class group in a qualitative formative study exploring children’s perceptions of the Bestlife physical activity app.

School and class group		Focus groups, n	Consenting pupils, n
Primary school 1			
	4	5	27
	6	1	6
Primary school 2			
	5a	1	5
	6a	1	4
	6b	3	18
Primary totals		11	60
Secondary school 1			
	7 to 9	1	3
Secondary school 2			
	7 to 9	1	5
Secondary totals		2	8
Overall totals		13	68

**Figure 1.**
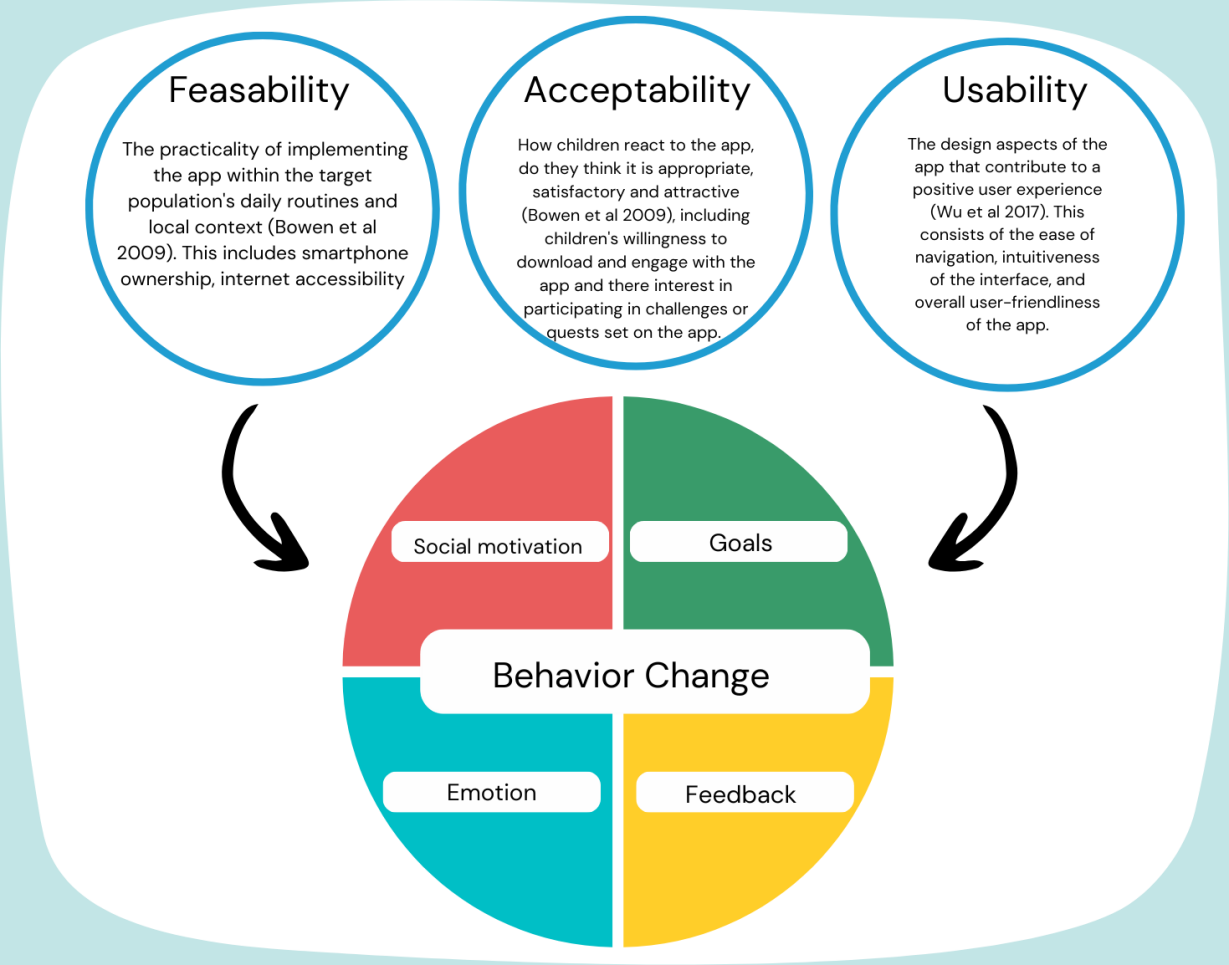
Conceptual framework for the evaluation of the Bestlife physical activity app [47,48].

The findings are presented through the perspectives of feasibility, usability, and acceptability, which collectively provide insights into the app’s overall effectiveness adoption (See Figure 1). These dimensions are intrinsically linked to the behavior change principles that were foundational to the app’s design. The factors of feasibility, usability, and acceptability help to examine the extent to which children and families engage positively with the app. These 3 dimensions influence and are influenced by behavior change techniques, such as goal setting (motivation), feedback (motivation), and social collaboration (opportunity), which were integrated into the app to encourage physical activity. The results section explores each dimension while highlighting how they support behavior change outcomes.

### Feasibility

This theme, identified through the process of inductive coding, covers the download process, social support, time pressures surrounding using the app, and the community and outside environment.

### The Download Process

Participants highlighted the app’s ease of download and its small file size as practical advantages. A secondary school student remarked, “Yeah, because it’s only a small download, like 38 megabytes” (Secondary school 1); this emphasizes its convenience, particularly in environments with limited storage or slow internet speeds. However, the requirement for providing a parental email during registration was perceived as a frustrating additional step in the process and a significant barrier to downloading the app, especially for older YCSs who preferred greater autonomy. As a participant noted:

*Having to have your parents [involvement] is another thing that just will add annoyance to downloading it.*

[Secondary school 1]

This suggests that streamlining the registration process to accommodate varying levels of independence for children accessing the app could enhance its appeal.

### Social Support

Social support from friends and family, such as playing with and helping one another, was mentioned by several children, for example:

*Mummy helps me with counting and doing the stopwatch.*

[Year 4, Primary school 1]

This was echoed by other children in year 4 who discussed family members helping them with the activities and the app. Whereas a child in year 6 (Primary school 1) mentioned involving a friend:

*Played it with my friend, she liked the skipping and being active.*

[Year 6, Primary school 1]

In addition to support from family and friends, children also valued the shared experience of using the app collaboratively. A participant mentioned, “It’s more fun to do the activities with someone, like when we shared the tablet” (Year 5, Primary school 2), suggesting that facilitating collaborative usage could further enhance the app’s appeal. These suggestions emphasize the value of integrating both collaborative and competitive features within the app to foster social engagement, which may in turn boost motivation and sustained use.

Furthermore, involving a community element such as linking to the “Junior Park Runs” (Secondary school 1) could increase social support beyond home and the classroom and therefore create a socially reinforced environment for behavior change.

### Connecting With the Local Area

The children mentioned several local areas they were familiar with that they would like added to the app where they could find QR codes. In addition, children mentioned using the app outside:

*I was in the park, and I was doing like hanging that thing, then I was doing star jumps, and then I had to do a little run.*

[Year 5, Primary school 2]

However, one child raised a concern that when they were in the park, they had “other things to be doing,” and when questioned further, they answered “like playing football or going on the slides” (Year 5, Primary school 2).

### Acceptability

This theme includes the children’s opinions of the overall app, the reward system, and customizable features. The types of activities and tutorials or support offered are also discussed. Children made positive comments about the app in general, such as “It encourages us to be active” and “decent activities” (Year 5, Primary school 2).

The app’s gamified features, such as points and rewards, were generally well-received and seen as motivating. A participant stated (child jumping up and down exclaiming), “We got 20 points!” (Primary school 2), with another expressing, “I like it because you get points” (Year 4, Primary school 1).

This emphasizes the immediate gratification experienced by children offered by the feedback within the app. YCSs also expressed a desire for proportional rewards based on the difficulty or physical demand of the activity, suggesting:

*10 or less, you should get 10 points; 10 to 20—you should get 20. And then if it’s 30 or more, then you can have 50.*

[Secondary school 1]

This highlights the potential for a more dynamic reward structure that reinforces individual effort on a sliding scale.

Customization was another important factor in acceptability. YCSs appreciated designing avatars—when you log into the app, you can choose a character and adapt their hair and skin color, clothes, shoes, and accessories. However, they emphasized the need for further personalization and representativeness, including culturally relevant options. A child noted:

*That’s one thing I’m like, where are the hijabs? This is for people in Bradford.*

[Secondary school 2]

This was echoed in another focus group where children asked for “A hijab,” ‘‘abaya,” and “more hijabs and jilbabs” (Year 5, Primary school 2).

Further options were requested in every focus group, including “a way to design your own avatar” (Year 5, Primary school 2).

Customization was seen positively, but more options were requested:

I liked that you could change clothes, but we need more colors and fun stuff like dresses or branded items like Nike.

[Primary school 1]

YCSs also requested the ability to add pet, sports equipment, and different accessories to their avatars, the focus group from Year 6 (Primary school 1) suggested: “Skipping ropes, hula hoops, footballs, basketballs” and the Year 6 (Primary school 2) adding “‘cats,’ ‘dogs,’ ‘birds,’ ‘snakes,’ and ‘pandas’” and a sample from Year 5 (Primary school 1) suggesting “*‘*Unicorns,’ ‘butterflies,’ ‘grasshoppers,’ ‘tigers,’ ‘cats,’ and ‘leopards’” and Year 5 (Primary school 2) focusing more on accessories such as: “‘headphones’ ‘golden guitar,’ ‘emerald chain,’ and ‘glasses.’”

These comments underline the importance of inclusive and diverse options in avatar design to cater to varied cultural, social, and creative preferences.

In addition, seasonal themes and event-based updates were requested in several focus groups. Suggestions such as “Maybe a light and dark mode or something like that” (Secondary school 1) and requests to “change the background” (Year 5, Primary school 2) reflect a desire for customizable visual settings. In addition, a participant highlighted the value of regular updates by stating*, “*Every so often it just needs to have an update to keep it being exciting” (Secondary school 2), while seasonal requests like “Christmas theme or summer outfits” (Year 5, Primary school 2) point to the potential for event-based content to maintain long-term engagement.

Participants proposed a diverse array of activities beyond traditional sports. Several children suggested physical activities such as “cooking and cleaning” (Year 6, Primary school 1) and even using a “5-minute timer to do a chore and you get points” (Year 5, Primary school 2), indicating that incorporating everyday tasks could enhance the app’s relevance. Similarly, for alternative sports-related activities, a participant noted*, “*If dance was added, I would have used the app a little bit more” (Secondary school 1), while others mentioned options like “20 minutes playing sports,” and activities, including “racing, gymnastics, dancing, jogging on the spot, football, traffic lights, basketball, dance to the beat, and cricket” (Year 5, Primary school 2). These suggestions highlight that a broader range of activity types could increase the app’s appeal and acceptability by catering to varied interests and daily routines.

To improve the acceptability of the app, YCSs suggested that accessing tutorial resources through the app would help. As one participant stated, “I don’t know how to do push-ups, but I tried. Maybe a tutorial or video would help” (Primary school 2). Providing instructional resources could enhance the children’s understanding of certain exercises.

Children’s emotional reactions to the app and its activities were multifaceted. For instance, one child remarked*, “*It just like reminds you how you feel when you do like different types of things” (Secondary school 1), while others used terms like “over the moon,” “overjoyed,” “tiring,” “exhausting,” and “ecstatic” (Years 6 and 4, Primary school). These varied responses suggest that while the app is engaging and can evoke positive emotions, some activities may also be perceived as physically demanding.

Social collaboration was also highlighted as a positive aspect. A participant shared:

*It’d be fun to see how I’m doing compared to my friends.*

[Year 6, Primary school 2]

This underscores the importance of fostering cooperative experiences to enhance the app’s appeal in social contexts.

Conversely, a child noted:

*We got 40 points! I like that we can compete with others to see who’s better.*

[Primary school 1]

### Usability

Topics covered under this theme include functionality, clarity of features, and app navigation. The app’s interface was generally described as intuitive and easy to navigate. A child noted referring to the activity dashboard—The activity dashboard is a simple graph of the number of activities completed and a step count over the week:

*It’s cool to see all the things I’ve done.*

[Secondary school 1]

However, some participants expressed confusion about certain features, which highlights the importance of including clearer terminology and guidance to support user navigation within the app. For instance, one participant stated, “When I went on, I didn’t know what ‘solo quest’ meant” (Secondary school 2) and suggested adding further guidance focused around how to use the app, though this was not supported by the other young people in the group. This potentially highlights the need for clearer explanations and guidance within the app to support user understanding.

YCSs also suggested adding a sound notification as an additional cue to mark the end of an activity, enhancing usability during active tasks:

*Something that they could watch and something that they could do whilst, whilst they were like, on their five, on like a five-minute run or something. Because otherwise they are just stood there running, watching their clock countdown.*

[Secondary school 1]

Collaborative features were appreciated for fostering competition and camaraderie, particularly in school settings. A child remarked:

*If you do it at school, there’s more people being competitive, like in a class.*

[Secondary school 1]

Emotional tracking refers to prompts that ask users to reflect on how they feel before and after engaging in physical activity, designed to encourage mindfulness about the emotional benefits of physical activity. Feedback on the emotion tracking feature varied, with some participants finding it valuable while others felt it was overused. A child explained, “It just like reminds you how you feel when you do like different types of things” (Secondary school 1 pupil), but they also suggested reducing its frequency to avoid frustration.

A participant also suggested that notifications could remind users to log their emotions even if they had not been active that day:

*If a notification popped up? Yeah, it’d make you remember to go on the app… like a lot of the time I won’t remember to go on any apps*

[Secondary school 2]

This indicates a need to balance the feature’s usability with user preferences. Participants also suggested other types of prompts to enhance engagement. For instance, a child proposed a “daily reward to make people want to play it more” (Secondary school 2), while another recommended that the app should ask, “Do you want to take a break now?” (Year 6, Primary school 2) if used for extended periods. These ideas point to the potential benefits of dynamic prompting to sustain user interest.

Suggestions for improving the app’s functionality focused on integration with existing health tools, which could align the app with user habits and enhance its utility. For example, a secondary school participant proposed linking the app to Samsung Health or Google Fitbit, stating:

*My phone with Samsung Health app automatically tracks my steps. If that was implemented* [integrated*], it would also see the steps.*

[Secondary school 1]

This feature would simplify tracking and reduce user effort, making the app easier to use and, therefore, appealing. Beyond step counting via Samsung Health, participants also expressed interest in additional biometric features. Comments such as “I want to track my heart rate” (Year 6, Primary school 1) indicate a demand for more comprehensive health monitoring. Furthermore, a participant queried*,* “You know the step activities, does the app track your steps?” (Year 4, Primary school 1).

Furthermore, several participants expressed a desire for multidevice compatibility. One noted, “In Bestlife I couldn’t do it on my laptop” (Year 6, Primary school 1), while another stated, “I want to do it on my Switch” (Year 6, Primary school 1). This suggests that extending support beyond smartphones may enhance usability by accommodating various user preferences.

Overall, improving clarity in the interface, integrating social and competitive features, and offering customizable options for auditory feedback would address key user needs and enhance the app’s usability and engagement.

### Behavior Change Techniques

The behavior change techniques theme highlights how Bestlife encourages engagement through goal-setting, external motivation, and reward-based reinforcement. The app prompts children to set clear activity goals via quests, which serve as external motivators and encourage physical activity.

*It’s like, fun to get points for doing activities like jumping jacks.*

[Primary school 2]

*I enjoyed doing the push-ups because it helps my body.*

[Year 4, Primary school 1]

These examples show how quests function both as motivational prompts and as tools for establishing behavioral goals.

Points earned from completing quests act as immediate, visible rewards, reinforcing children’s efforts and contributing to sustained engagement.

*We got 20 points!*

[Primary school 2]

Other children expressed that effort should be rewarded proportionately:

*If someone worked harder, they should get more points.*

[Year 6, Primary school 1]

*More points for doing jumping jacks than walking.*

[Secondary school 2]

These comments suggest that a graded reward system could enhance fairness and motivation. While many children valued these external incentives, some also described intrinsic enjoyment in physical activity itself:

*Riding my bike is fun, I don’t need points for that.*

[Secondary school 1]

The app also includes emotional tracking before and after activity to encourage self-reflection. However, children had mixed views about its frequency:

*Once a day would be better.*

[Secondary school 2]

*Maybe like one before you do a full quest and then one after.*

[Secondary school 1]

*Don’t ask again button.*

[Year 5, Primary school 2]

These responses suggest that reducing the frequency or offering flexible options could enhance the user experience while preserving the reflective benefits.

Social support emerged as a key driver of engagement. The “collab mode” allows users to team up with friends or classmates, enhancing motivation through shared goals.

*It’s good because you can collab with your friends.*

[Primary school 2]

Social influences extended beyond peers. A participant remarked:

*I’d probably end up doing it if my mum nagged at me hard enough.*

[Secondary school 1]

Competitive dynamics, particularly in school settings, also contributed:

*If you do it at school, there’s more people being competitive, like in a class.*

[Secondary school 2]

A child suggested linking the app to household tasks:

*It would persuade you to do it. I’m not going to lie I’m quite lazy when my mum says go do your chores [name]. So it could be a way to persuade. Competitiveness with yourself.*

[Year 5, Primary school 2]

Progress tracking features, such as the activity dashboard, enabled children to monitor achievements and maintain motivation. Children set specific, measurable goals:

*Let’s see if we got more than 20.*

[Year 4, Primary school 1]

*We set a goal to do 1000 steps a day.*

[Secondary school 1]

Longer-term goals also played a role:

*Trying to reach the next level made me keep going.*

[Secondary school 2]

Reflecting on progress, a child shared:

*It’s cool to see all the things I’ve done.*

[Secondary school 1]

Another added:

*I liked seeing how much I did and comparing it with others.*

[Primary school 1]

To sustain engagement, a participant emphasized the importance of variety:

*Every so often it just needs to have an update to keep it being exciting.*

[Secondary school 2]

In summary, the app effectively integrates several behavior change techniques, including goal-setting, rewards, social support, and self-monitoring. Participant feedback suggests that while these features support engagement, refinements, such as adjusting emotional tracking frequency and personalizing reward systems, could further enhance the app’s effectiveness and appeal.

## Discussion

### Principal Findings

**Figure 2.**
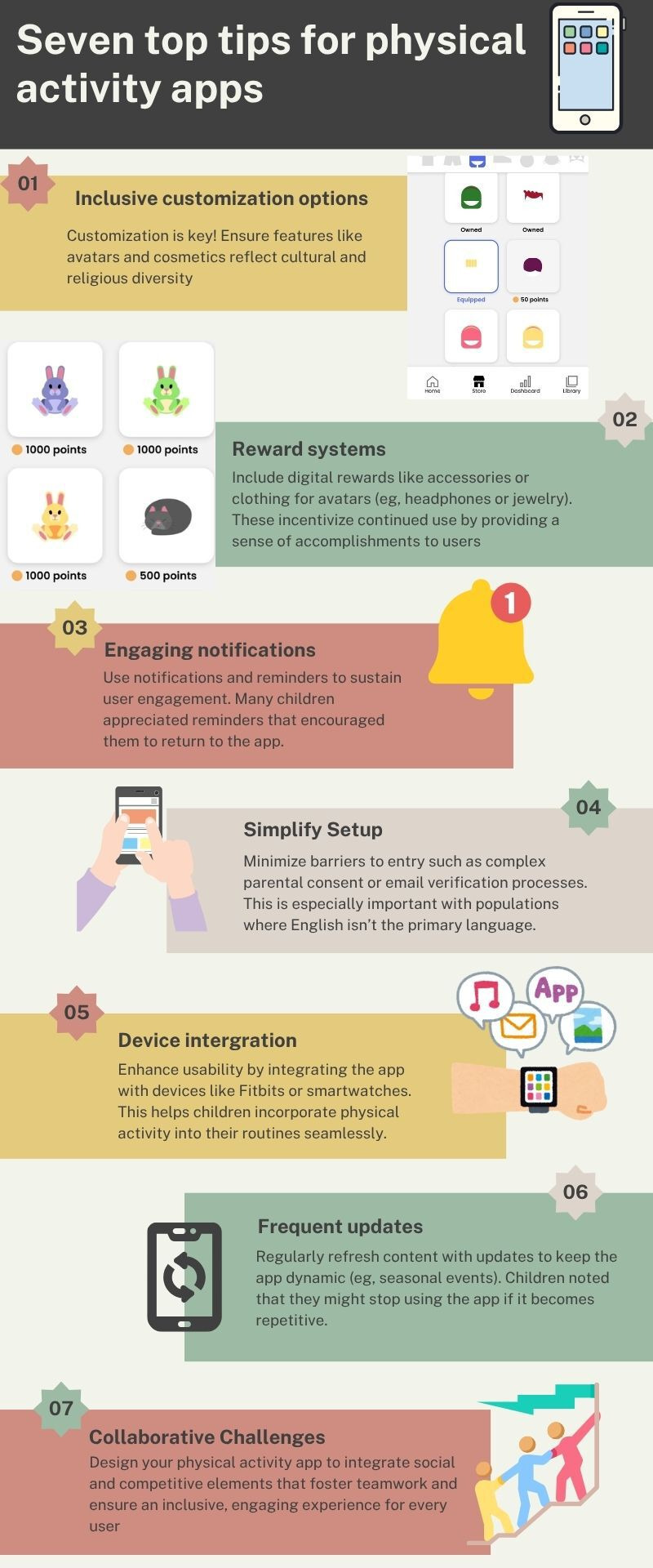
Evidence-based recommendations for designing physical activity apps for children.

This study found that the Bestlife app was generally feasible, acceptable, and user-friendly for children aged 8-13 years. Positive engagement was particularly driven by gamified features, social collaboration, and extensive personalization options. Notably, children responded positively to features enabling self-monitoring, goal-setting, and proportional rewards, while highlighting the importance of aligning digital health tools with children’s daily routines and social contexts. While some findings confirm established insights from previous research, our study extends the literature by using a citizen science methodology that actively involved children as co-evaluators, uncovering specific barriers, such as the parental email registration requirement, that could limit autonomy and engagement among older children. Furthermore, children’s explicit requests for culturally inclusive customization, including diverse clothing and accessories, provide novel and actionable insights for culturally sensitive app development. These findings not only inform ongoing refinements of the Bestlife app but also offer broader, child-driven recommendations for future mHealth interventions targeting diverse youth populations (see Figure 2).

### Feasibility

Our findings indicate that implementing the Bestlife app was generally feasible in a real-world setting. Children reported that the app’s small download size and easy installation facilitated uptake even on devices with limited storage or bandwidth. This practical design choice helped avoid technical barriers that often hinder mHealth adoption in low-resource environments. Previous research similarly emphasizes that minimizing technical demands (eg, download size, bugs, etc) is crucial for user retention [60-62]. Our study uniquely highlights how a small app size, when combined with familiar contexts such as school-based rollout, may mitigate infrastructure-related barriers, a factor that remains underexplored in current mHealth literature targeting children and young people. By ensuring Bestlife ran reliably on basic devices, the developers addressed a key feasibility concern identified both in our study and the literature. This insight was uncovered through a participatory citizen science approach, where children acted not just as users but as co-evaluators of the intervention. Their feedback, gathered through real-world app use in everyday settings, strengthens the validity of these findings and surfaces nuanced design implications that might be missed in traditional researcher-led evaluations.

Despite these strengths, our participants revealed a procedural hurdle: the mandatory parental email registration. Older children in particular found the required parental sign-up frustrating, as it delayed or limited their independent access to the app’s features. While parental oversight is important for ensuring children’s safety, our findings suggest that this requirement inadvertently discouraged some users. This is consistent with observations where mandatory parental consent for app use and research participation can act as a barrier, particularly for adolescents seeking real-time engagement with digital tools [63]. Simplifying the sign-up process or offering age-appropriate alternatives (such as using school credentials) could improve feasibility without compromising child safety [63,64]. Streamlining the onboarding experience is a clear design implication from our study: future child-focused apps should balance necessary parental controls with a frictionless entry for young users.

The time constraints children face were another barrier to feasibility. Children have busy schedules, and some struggled to fit app sessions into their daily routine. In our focus groups, a few children mentioned that homework, chores, or other activities left little time for using Bestlife regularly. To address this, participants discussed integrating app activities into existing routines, for example, incorporating short activity challenges that would monitor chores. This finding suggests that making the app complement rather than compete with daily activities would enhance its feasibility for sustained use. Embedding health prompts into everyday life is known to support habit formation [45]. Consistent with this idea, research indicates that aligning interventions with children’s usual schedules (eg, walking to school or family playtime) increases engagement [65]. Future designs should therefore consider seamless integration into children’s routines.

### Acceptability

Children found the Bestlife app to be acceptable overall, and they were enthusiastic about its gamified features and theme. Children responded positively to the app’s points, rewards, and activity elements, which made being physically active fun. The participants described different ways gamification features like earning points for completing exercises motivated them, indicating that these elements successfully tapped into their sense of play. This aligns with previous findings that game-like rewards and feedback can boost engagement in youth physical activity interventions [35,66]. In the Step It Up Family program, for instance, competitive step challenges and leaderboards were key motivators for kids and parents alike [35]. However, our citizen science data offer new insight into how children prefer collaborative over competitive play, suggesting that app-based motivation may be more sustainable when framed as team success rather than individual rivalry.

The Bestlife users similarly enjoyed friendly competition, such as comparing points in collab challenges; but importantly, many expressed a preference for collaboration over pure competition. Rather than outdoing friends, children said they loved team-based challenges and shared participation, especially when used in their classroom groups. This is a critical insight: while competition can spur short-term engagement, collaborative play created enjoyment and fostered a sense of community. Past research supports this, noting that cooperation and social support can enhance motivation without the downsides of rivalry [23,31]. These findings suggest that mHealth interventions targeting children may benefit from prioritizing peer collaboration to foster inclusivity, especially within school environments.

Cultural relevance and inclusivity were also highlighted as important. Children in diverse Bradford schools appreciated Bestlife’s avatar customization but also pointed out its limited options reflecting different cultures and identities. For example, some girls noted the lack of modest clothing or hijab options for avatars, and others wished for attire or accessories that resonated with their cultural background. This extends existing literature by providing grounded, child-led evidence that superficial customization is insufficient, children expect inclusive representation. When children cannot create an avatar that looks like them or reflects their lifestyle, the app may feel less welcoming. Our data underscore the value of inclusive design: several children explicitly suggested adding a wider range of skin tones, clothing styles, and accessories. In the broader literature, inclusivity in game design has been linked to greater user identification and satisfaction [67]. Providing diverse options and culturally sensitive content can make health apps more appealing to underserved groups [68-70]. Our findings support this and additionally offer actionable design recommendations: modest sportswear, culturally relevant hairstyles, and seasonal or religious festival themes could meaningfully improve acceptability in multiethnic youth populations.

Participants also gave feedback on how to keep the app interesting and acceptable over the long term. A common suggestion was to introduce customizable notifications and dynamic content updates. Children liked the idea of gentle reminders, for instance, a smartphone notification nudging them to do a quick activity or themed holiday linked reminders. This aligns with evidence that regular prompts can re-engage users and sustain healthy behaviors [66,71]. Importantly, the tone and timing of these reminders must be child-friendly; generic text-message prompts used in adult interventions have shown limited impact on children [35].

### Usability

The usability of the Bestlife app was generally well-received, with most children finding it easy to navigate and use. This intuitive navigation is a strong point of the app’s design. It suggests that even first-time users were able to explore features independently, which is crucial for mHealth interventions targeting children. Previous studies note that ease of use significantly influences whether young people keep using an app [72,73]. Our findings reinforce that principle: a child-friendly interface, with straightforward menus, enabled engagement without frustration. Notably, conducting focus groups in classroom settings allowed us to observe that many children required little help from adults or peers to use Bestlife, indicating a high level of usability in its current form.

Despite this overall positive feedback, our study also uncovered challenges for certain users, highlighting the need to accommodate varying levels of digital literacy among children. A subset of the participants less familiar with smartphones struggled with some of the in-app terminology and task flow. For instance, a few children were confused by labels like or did not immediately understand how to progress from one challenge to the next without guidance. These instances show that what older or more tech-savvy children find intuitive might not be obvious to all users.

Our data suggest a balanced solution: include optional tutorials and visual guides for those who need them. Several children in the focus groups said they would have used a short tutorial video if available, whereas others felt they did not need it. Providing an on-demand tutorial (for example, a friendly animated guide explaining how to use the app) could support less digitally literate users without forcing mandatory instruction on those who prefer to learn by exploration. This approach echoes design practices in other child-oriented apps; for example, the Jungle Gym (Learning Games Lab) physical activity app introduced a toggleable guide for new users, which improved usability for younger children while older kids ignored it once they felt confident [61]. In line with previous recommendations on digital health literacy [74-76], we recommend that future mHealth apps for youth conduct age-specific usability testing to ensure interface text and navigation are easily understood by the youngest users. By refining terminology and offering help, Bestlife and similar apps can become truly accessible to children of all ages and skill levels.

Overall, our findings underline that usability is not one-size-fits-all for children. Successful apps must cater to a range of ages and capabilities, providing simplicity for novices and depth for experienced users. We recommend that future mHealth interventions adopt a layered usability model, offering core functionality up front with optional depth and scaffolding for those who need it. By embracing inclusive design and iterative usability testing, future interventions can ensure that technical usability issues do not become a barrier to health engagement and equity.

### Behavior Change

Our qualitative evidence suggests that the Bestlife app incorporated several behavior change techniques (BCTs) that resonated with children and showed promise for encouraging physical activity. Children mentioned features such as setting personal goals, monitoring their progress, and sharing achievements with friends, all of which are well-established strategies in behavior change theory [45]. For example, goal-setting and self-monitoring were embedded within Bestlife through activity challenges and dashboards that visually displayed points earned. These features effectively tapped into different motivational drivers: some children expressed intrinsic motivation derived from personal mastery and achievement, such as experiencing pride in improving their personal records (eg, completing more push-ups than previously), while others highlighted extrinsic motivation through social recognition and tangible rewards, such as points and avatar upgrades [77]. Distinguishing these motivational pathways provides valuable insights for future interventions, suggesting tailored features to cater separately or simultaneously to intrinsic and extrinsic motivations to maximize engagement and sustainability.

Feedback mechanisms within Bestlife also significantly contributed to promoting behavior change. The app’s well-being tracker enabled children to log mood or energy levels, enhancing awareness of the emotional benefits associated with physical activity. Some participants explicitly noted connections between activity and mood improvements, describing physical challenges as “fun.” Such reflective feedback reinforces behavior change by validating efforts and encouraging continued participation [45]. Future iterations of mHealth apps should continue integrating feedback loops, which clearly connect children’s physical activities with emotional outcomes [40].

### Implications for Future Research and Practice

The iterative development process based on user feedback was a significant strength of this study. By directly engaging with children and incorporating their suggestions such as higher-value store items, modest clothing options, and tutorial videos, the app evolved in real time to better meet the needs and preferences of its target users. These iterative updates not only enhanced usability but also ensured that the app’s features were grounded in evidence supporting the modeling of behavior change in physical activity interventions [26]. This study clearly demonstrates the critical value of a participatory design approach, where children’s voices drive the evolution of the intervention. We strongly recommend that future app development projects adopt a similar iterative co-design process, as it fosters responsive, user-centered design and ultimately leads to more engaging and effective digital interventions.

While Bestlife seeks to address physical inactivity among young people through engaging digital media, it is essential to critically examine the broader implications of promoting smartphone use in this age group. Increasing advocacy for delaying smartphone ownership until 16 years of age reflects growing concerns about the risks to mental health and online safety associated with early digital engagement [78,79]. Research suggests that early and prolonged smartphone exposure may contribute to poor mental well-being, cyberbullying exposure, and reduced real-world social interactions [17,80]. These concerns raise an important ethical question: by creating attractive, gamified content aimed at young users, are we unintentionally reinforcing or accelerating problematic digital behaviors? To mitigate these risks, the deployment of Bestlife must continue to be guided by a framework that includes parental oversight, clear usage boundaries, and an emphasis on balance between digital and offline activities [21,81], ensuring we support and not compromise young people’s long-term well-being.

The findings of this study contribute to the growing evidence base for child-focused mHealth interventions. They underscore the importance of integrating behavior change techniques with user-centered design principles while addressing practical barriers to accessibility and engagement. Future physical activity apps designed for children should prioritize inclusive customization options, reward systems, engaging notifications, simplifying the setup process, device integration, collaborative challenges, and frequent updates (see Figure 2).

Future research should focus on evaluating the app’s long-term impact on physical activity to see if it is still being used and to assess children’s behavior change, and exploring its scalability in diverse cultural and socioeconomic contexts. By situating the findings within the broader landscape of mHealth research, this study highlights the potential of well-designed digital tools to promote physical activity among children while addressing the complexities of behavior change.

### Strengths and Limitations

This study offers valuable insights into the design and evaluation of a child-focused mHealth app, underpinned by several key strengths. The use of a contributory citizen science approach, where children acted as active co-evaluators rather than passive participants, ensured that findings were deeply rooted in lived experience. Children trialed the app in real-world settings and provided direct feedback, shaping both the evaluation and the app’s ongoing development. This participatory model not only supports authentic engagement but also enables iterative adaptation of app features in response to children’s feedback, such as requests for more diverse avatar customization options and higher-value in-app rewards.

However, the collaborative school-based setting that made this feedback-rich approach possible also introduces potential bias. Group-based focus sessions created an enthusiastic and supportive environment, which may have inflated perceived engagement compared to independent use at home. While peer encouragement likely encouraged more expressive and positive feedback, it may not fully reflect solitary user experiences. Future studies could compare app engagement in individual versus group contexts to more clearly understand this dynamic.

Another notable strength was the diverse participant sample, spanning a wide age range (8‐13 y) and including children from multiethnic, socioeconomically varied backgrounds in Bradford, a city with high levels of childhood inactivity and digital inequality. Capturing the perspectives of these underrepresented groups enhances the relevance of our findings for public health. Nonetheless, some sampling limitations were present. Due to the need for active parental consent, not all children who used the app were included in the formal research sample. This may have skewed participation toward families already more engaged in school or research activities, limiting representativeness.

The study’s embeddedness in familiar school environments was another strength that contributed to open, honest dialog. Conducting focus groups in classrooms, often with trusted teachers nearby, fostered comfort and richer interaction. Yet, this embedded approach also constrained the scope of behavioral observation. Children used the app for a relatively short period (1‐2 weeks), which limited our ability to assess sustained behavior change or long-term retention. While early indicators of motivation and habit formation were promising, and children themselves proposed features (like reminders and content updates) to support longer engagement, we were not able to evaluate these strategies over time. A longitudinal design, paired with app analytics, would strengthen future evaluations.

Ethically, the study upheld high standards of informed consent and child autonomy, for instance, respecting a child’s choice to withdraw despite parental permission. Still, we recognize that requiring written parental consent can restrict inclusion, particularly in more marginalized families. In future low-risk studies of this nature, opt-out consent models could help broaden participation while maintaining ethical safeguards.

Finally, while the study offers actionable learning for mHealth development, its findings are inevitably shaped by the specific cultural and geographic context of one UK city. Bestlife was tailored for use in Bradford, and although many design recommendations are broadly transferable, further testing in other locations and demographics is needed to assess generalizability.

Despite these limitations, the study provides credible, grounded, and highly applicable insights for those designing digital interventions for children. The emphasis on children’s voices throughout enhances the study’s relevance, and the real-world setting lends practical depth that many controlled evaluations lack. These findings lay a strong foundation for future research and development of inclusive, engaging, and behaviorally effective digital tools to support children’s health.

### Conclusion

As digital health tools increasingly shape the health behaviors of children, understanding what truly resonates with young users has never been more critical. This formative research contributes valuable insight to the limited literature on children’s experiences with physical activity apps. By exploring user perceptions of app features, content, and usability, this study identified key design elements that enhance engagement such as gamification and opportunities for collaboration. These findings not only inform the ongoing refinement of the Best Life app but also offer practical guidance for future developers aiming to design child-centered, culturally inclusive digital interventions. In addition, this research provides a foundation for using such apps to both promote physical activity and collect meaningful data on young people’s health behaviors in real-world contexts.

## Supplementary material

10.2196/76498 Multimedia Appendix 1 How to play.

10.2196/76498 Multimedia Appendix 2 Booklet for citizen scientists to complete at home.

10.2196/76498 Multimedia Appendix 3 Focus group guide.

10.2196/76498 Multimedia Appendix 4 Code book.

10.2196/76498 Checklist 1 COREQ checklist.

## Abbreviations

BCW: Behavior Change Wheel
JU:MP: Join Us: Move. Play
mHealth: mobile health
YCS: Young Citizen Scientist

## References

[1] Bailey R, Hillman C, Arent S, Petitpas A (2013). Physical activity: an underestimated investment in human capital?. J Phys Act Health.

[2] (2024). Physical activity. WHO.

[3] Eyre ELJ, Duncan MJ (2013). The impact of ethnicity on objectively measured physical activity in children. ISRN Obes.

[4] Love R, Adams J, van Sluijs EMF, Foster C, Humphreys D (2018). A cumulative meta-analysis of the effects of individual physical activity interventions targeting healthy adults. Obes Rev.

[5] Dumith SC, Gigante DP, Domingues MR, Kohl HW, Kohl HW (2011). Physical activity change during adolescence: a systematic review and a pooled analysis. Int J Epidemiol.

[6] Bagnall AM, Radley D, Jones R (2019). Whole systems approaches to obesity and other complex public health challenges: a systematic review. BMC Public Health.

[7] Telama R, Yang X, Viikari J, Välimäki I, Wanne O, Raitakari OT (2005). Physical activity from childhood to adulthood: a 21-year tracking study. Am J Prev Med.

[8] (2023). Active lives children and young people survey - academic year 2022-23.

[9] Ryan D, Nutting H, Parekh C (2024). Ready, set, co(produce): a co-operative inquiry into co-producing research to explore adolescent health and wellbeing in the Born in Bradford Age of Wonder project. Res Involv Engagem.

[10] Bull FC, Al-Ansari SS, Biddle S (2020). World Health Organization 2020 guidelines on physical activity and sedentary behaviour. Br J Sports Med.

[11] WHO Global Observatory for eHealth (2011). mHealth: new horizons for health through mobile technologies: second global survey on eHealth. WHO.

[12] (2023). Children and parents: media use and attitudes report 2023. Ofcom.

[13] Casey A, Goodyear VA, Armour KM (2017). Rethinking the relationship between pedagogy, technology and learning in health and physical education. Sport Educ Soc.

[14] Greenhow C, Lewin C (2016). Social media and education: reconceptualizing the boundaries of formal and informal learning. Learn Media Technol.

[15] Seah MLC, Koh KT (2021). The efficacy of using mobile applications in changing adolescent girls’ physical activity behaviour during weekends. Eur Phy Educ Rev.

[16] Kemp BJ, Parrish AM, Cliff DP (2020). “Social screens” and “the mainstream”: longitudinal competitors of non-organized physical activity in the transition from childhood to adolescence. Int J Behav Nutr Phys Act.

[17] Abi-Jaoude E, Naylor KT, Pignatiello A (2020). Smartphones, social media use and youth mental health. CMAJ.

[18] Pires RCR, Reinert A, Cantanhede AM, Pinheiro J (2024). Associations between smartphone use, smartphone addiction and mental health in teenagers: a structural equation modeling approach. R Tecnol Soc.

[19] Caninsti R, Rahayu SAT (2024). The role of smartphone addiction on bed procrastination and mindful eating behavior in adolescents. indigenous.

[20] Tater B, John K (2024). Effects of smartphone addiction on the physical and mental well-being of indian students. IJST.

[21] Mohta R, Halder S (2021). A comparative study on cognitive, emotional, and social functioning in adolescents with and without smartphone addiction. JIACAM.

[22] Mollborn S, Limburg A, Pace J, Fomby P (2022). Family socioeconomic status and children’s screen time. J Marriage Fam.

[23] Sharma S, Gergen Barnett K, Maypole JJ, Grochow Mishuris R (2022). Evaluation of mHealth apps for diverse, low-income patient populations: framework development and application study. JMIR Form Res.

[24] Sohn SY, Rees P, Wildridge B, Kalk NJ, Carter B (2019). Prevalence of problematic smartphone usage and associated mental health outcomes amongst children and young people: a systematic review, meta-analysis and GRADE of the evidence. BMC Psychiatry.

[25] Emberson MA, Lalande A, Wang D, McDonough DJ, Liu W, Gao Z (2021). Effectiveness of smartphone-based physical activity interventions on individuals’ health outcomes: a systematic review. Biomed Res Int.

[26] Conroy DE, Yang CH, Maher JP (2014). Behavior change techniques in top-ranked mobile apps for physical activity. Am J Prev Med.

[27] Bezabih AM, Gerling K, Abebe W, Abeele VV (2021). Behavioral theories and motivational features underlying ehealth interventions for adolescent antiretroviral adherence: systematic review. JMIR Mhealth Uhealth.

[28] Bort-Roig J, Gilson ND, Puig-Ribera A, Contreras RS, Trost SG (2014). Measuring and influencing physical activity with smartphone technology: a systematic review. Sports Med.

[29] Ford KL, Moore SL, Zhou S, Gore MO, Portz J, Zhang X (2019). Advancing evidence-based digital health through an innovative research environment: an academic-industry collaboration case report. mHealth.

[30] Wang Y, Wang Y, Greene B, Sun L (2020). An analysis and evaluation of quality and behavioral change techniques among physical activity apps in China. Int J Med Inform.

[31] Krouwel M, Greenfield SM, Chalkley A (2023). Promoting participation in physical activity through Snacktivity: A qualitative mixed methods study. PLoS ONE.

[32] Brannan MGT, Foster CE, Timpson CM (2019). Active 10 - A new approach to increase physical activity in inactive people in England. Prog Cardiovasc Dis.

[33] Liang H, Wang X, An R (2023). Influence of Pokémon GO on physical activity and psychosocial well-being in children and adolescents: systematic review. J Med Internet Res.

[34] Schoffman DE, Turner-McGrievy G, Jones SJ, Wilcox S (2013). Mobile apps for pediatric obesity prevention and treatment, healthy eating, and physical activity promotion: just fun and games?. Transl Behav Med.

[35] Schoeppe S, Salmon J, Williams S (2022). Feasibility of using activity trackers and apps to increase physical activity in whole families: The Step it Up Family intervention. Digit Health.

[36] Odenigbo IP, Reen JK, Eneze C, Friday A, Orji R Virtual, augmented, and mixed reality interventions for physical activity: a systematic review. http://www.scopus.com/inward/record.url?scp=85146227420&partnerID=8YFLogxK.

[37] Gkintoni E, Vantaraki F, Skoulidi C, Anastassopoulos P, Vantarakis A (2024). Promoting physical and mental health among children and adolescents via gamification-a conceptual systematic review. Behav Sci (Basel).

[38] Tong HL, Coiera E, Laranjo L (2018). Using a mobile social networking app to promote physical activity: a qualitative study of users’ perspectives. J Med Internet Res.

[39] Simons D, De Bourdeaudhuij I, Clarys P, De Cocker K, Vandelanotte C, Deforche B (2018). A smartphone app to promote an active lifestyle in lower-educated working young adults: development, usability, acceptability, and feasibility study. JMIR Mhealth Uhealth.

[40] Hosseinpour M, Terlutter R (2019). Your personal motivator is with you: a systematic review of mobile phone applications aiming at increasing physical activity. Sports Med.

[41] Wang JW, Zhu Z, Shuling Z (2024). Effectiveness of mHealth app–based interventions for increasing physical activity and improving physical fitness in children and adolescents: systematic review and meta-analysis. JMIR Mhealth Uhealth.

[42] Western MJ, Armstrong MEG, Islam I, Morgan K, Jones UF, Kelson MJ (2021). The effectiveness of digital interventions for increasing physical activity in individuals of low socioeconomic status: a systematic review and meta-analysis. Int J Behav Nutr Phys Act.

[43] Whitehead L, Talevski J, Fatehi F, Beauchamp A (2023). Barriers to and facilitators of digital health among culturally and linguistically diverse populations: qualitative systematic review. J Med Internet Res.

[44] (2023). What is JU:MP?. Active Bradford.

[45] Michie S, van Stralen MM, West R (2011). The behaviour change wheel: A new method for characterising and designing behaviour change interventions. Implementation Sci.

[46] Born in Bradford. Bradford Institute for Health Research.

[47] Bowen DJ, Kreuter M, Spring B (2009). How we design feasibility studies. Am J Prev Med.

[48] Wu J, Tombor I, Shahab L, West R (2017). Usability testing of a smoking cessation smartphone application ('SmokeFree Baby’): A think-aloud study with pregnant smokers. Digit HEALTH.

[49] Bonney R, Cooper CB, Dickinson J (2009). Citizen science: a developing tool for expanding science knowledge and scientific literacy. Bioscience.

[50] (2022). Ten principles of citizen science. ECSA.

[51] Shirk JL, Ballard HL, Wilderman CC (2012). Public participation in scientific research: a framework for deliberate design. E&S.

[52] Wargers A, Queral J, Mölenberg FJ (2023). Citizen Science to improve healthy and active living among adolescents in four European countries: a protocol of the cluster randomised controlled trial of the Science Engagement to Empower aDolescentS (SEEDS) project. BMJ Open.

[53] Frazer M, Seims A, Tatterton MJ (2023). Child and family experiences of a whole-systems approach to physical activity in a multiethnic UK city: a citizen science evaluation protocol. BMJ Open.

[54] Frazer MT, Creaser A, Tatterton MJ, Daly-Smith A, Hall J (2024). Exploring children and young people’s experience of participating in citizen science-A qualitative evidence synthesis. PLOS ONE.

[55] Ballard HL, Dixon CGH, Harris EM (2017). Youth-focused citizen science: Examining the role of environmental science learning and agency for conservation. Biol Conserv.

[56] Bender K, Barman-Adhikari A, DeChants J (2017). Asking for change: feasibility, acceptability, and preliminary outcomes of a manualized photovoice intervention with youth experiencing homelessness. Child Youth Serv Rev.

[57] Lane HG, Porter KJ, Hecht E, Harris P, Zoellner JM (2019). A participatory process to engage appalachian youth in reducing sugar-sweetened beverage consumption. Health Promot Pract.

[58] Adler K, Salanterä S, Zumstein-Shaha M (2019). Focus group interviews in child, youth, and parent research: an integrative literature review. Int J Qual Methods.

[59] Noonan RJ, Boddy LM, Fairclough SJ, Knowles ZR (2016). Write, draw, show, and tell: a child-centred dual methodology to explore perceptions of out-of-school physical activity. BMC Public Health.

[60] Smoll NR, Walker J, Khandaker G (2021). The barriers and enablers to downloading the COVIDSafe app - a topic modelling analysis. Aust N Z J Public Health.

[61] McCloskey M, Johnson SL, Benz C (2018). Parent perceptions of mobile device use among preschool-aged children in rural head start centers. J Nutr Educ Behav.

[62] (2022). New IDS research shows poorest households trapped in digital poverty cycle. Institute of Development Studies.

[63] Badillo-Urquiola K, Chouhan C, Chancellor S, De Choudhary M, Wisniewski P (2020). Beyond parental control: designing adolescent online safety apps using value sensitive design. J Adolesc Res.

[64] Baumel A, Muench F, Edan S, Kane JM (2019). Objective user engagement with mental health apps: systematic search and panel-based usage analysis. J Med Internet Res.

[65] McKay FH, Wright A, Shill J, Stephens H, Uccellini M (2019). Using health and well-being apps for behavior change: a systematic search and rating of apps. JMIR Mhealth Uhealth.

[66] Borghouts J, Eikey E, Mark G (2021). Barriers to and facilitators of user engagement with digital mental health interventions: systematic review. J Med Internet Res.

[67] Alqahtani F, Winn A, Orji R (2021). Co-designing a mobile app to improve mental health and well-being: focus group study. JMIR Form Res.

[68] LeSeure P, Chin E, Zhang S (2024). A culturally sensitive mobile app (DiaFriend) to improve self-care in patients with type 2 diabetes: development study. JMIR Diabetes.

[69] Povey J, Mills P, Dingwall KM (2016). Acceptability of mental health apps for Aboriginal and Torres Strait Islander Australians: a qualitative study. J Med Internet Res.

[70] Garnweidner-Holme L, Hoel Andersen T, Sando MW, Noll J, Lukasse M (2018). Health care professionals’ attitudes toward, and experiences of using, a culture-sensitive smartphone app for women with gestational diabetes mellitus: qualitative study. JMIR Mhealth Uhealth.

[71] Fry JP, Neff RA (2009). Periodic prompts and reminders in health promotion and health behavior interventions: systematic review. J Med Internet Res.

[72] Russell E, Lloyd-Houldey A, Memon A, Yarker J (2018). Factors influencing uptake and use of a new health information app for young people. J Technol Hum Serv.

[73] Goodyear VA, Armour KM (2018). Young people’s perspectives on and experiences of health-related social media, apps, and wearable health devices. Soc Sci (Basel).

[74] Ridout B, Campbell A (2018). The use of social networking sites in mental health interventions for young people: systematic review. J Med Internet Res.

[75] Geng L, Jiang G, Yu L (2023). The most popular commercial weight management apps in the Chinese app store: analysis of quality, features, and behavior change techniques. JMIR Mhealth Uhealth.

[76] Baretta D, Perski O, Steca P (2019). Exploring users’ experiences of the uptake and adoption of physical activity apps: longitudinal qualitative study. JMIR Mhealth Uhealth.

[77] Ryan RM, Deci EL (2000). Intrinsic and extrinsic motivations: classic definitions and new directions. Contemp Educ Psychol.

[78] Twenge JM, Campbell WK (2018). Associations between screen time and lower psychological well-being among children and adolescents: Evidence from a population-based study. Prev Med Rep.

[79] Odgers CL, Jensen MR (2020). Annual Research Review: Adolescent mental health in the digital age: facts, fears, and future directions. Child Psychology Psychiatry.

[80] George MJ, Odgers CL (2015). Seven fears and the science of how mobile technologies may be influencing adolescents in the digital age. Perspect Psychol Sci.

[81] Livingstone S, Ólafsson K, Helsper EJ, Lupiáñez-Villanueva F, Veltri GA, Folkvord F (2017). Maximizing opportunities and minimizing risks for children online: the role of digital skills in emerging strategies of parental mediation. J Commun.

