# Supplementary material for: Active Play in a Digital Age, Exploring Children’s (Aged 8-13 Years) Views of a Physical Activity App: Qualitative Formative Study
Source: JMIR Form Res. 2025 Nov 11;9:e76498. doi: 10.2196/76498 (PMC12614868; doi:10.2196/76498)
Supplement: Multimedia Appendix 1 [file formative-v9-e76498-s001.docx]

**Appendix 1: How to play**

**Bestlife App**

### **Key Features:**

· **Engagement with Physical Activity:** Challenges and rewards to encourage participation.

· **Emotional Wellbeing:** Tracks emotional wellbeing alongside physical activity.

· **Gamification:** Digital rewards for completing challenges (e.g., standing on one leg, running around a local park).

· **Personalisation:** Children can choose avatars, complete quests, and earn points to spend on virtual items for their avatars.

**How to play:**

| 1) Choose an avatar  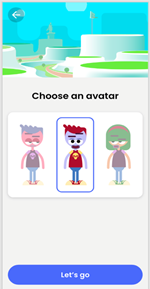 | 2) Choose a challenge to complete to earn points (solo or collaboratively)  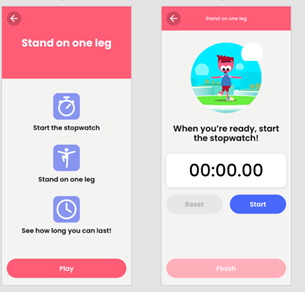 |
| --- | --- |
| 3) Quests can be completed  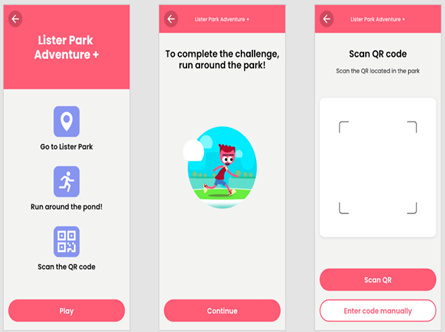 | 4) Upon completion of quest/challenge points are awarded that can be spend on clothes/hair etc for the avatar  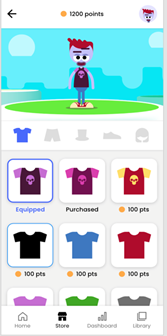 |
| 5) Before and after a quest or challenge feelings can be recorded on a scale  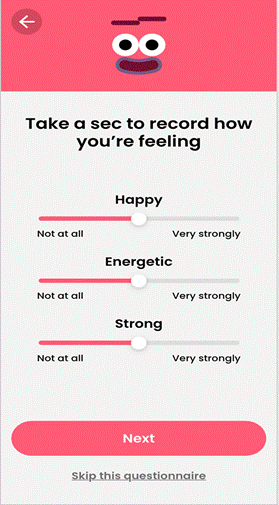 | 6) Dashboards monitor physical activity and well-being over time  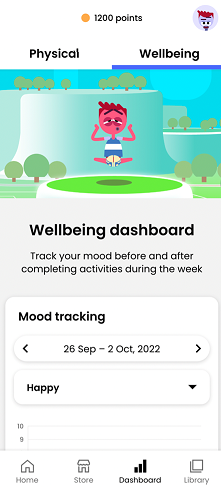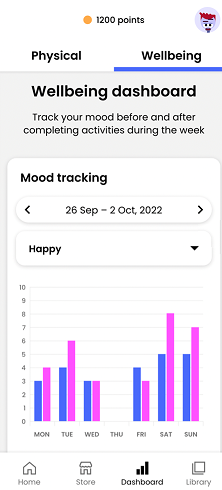 |
