# Supplementary material for: Active Play in a Digital Age, Exploring Children’s (Aged 8-13 Years) Views of a Physical Activity App: Qualitative Formative Study
Source: JMIR Form Res. 2025 Nov 11;9:e76498. doi: 10.2196/76498 (PMC12614868; doi:10.2196/76498)
Supplement: Multimedia Appendix 2 [file formative-v9-e76498-s002.pdf]

CITIZEN SCIENCE

JU:MP

bestlife

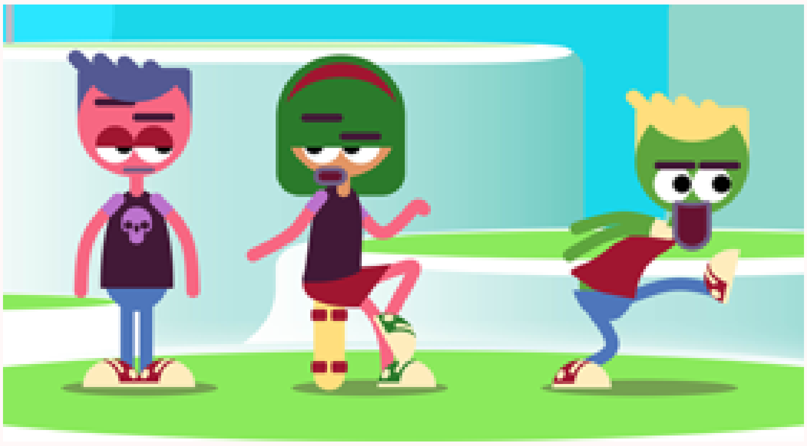

Remember to bring this back to school on

so we can talk about bestlife.

School: ....

Year: ....

What is a citizen scientist?

Someone like you who wants to create new knowledge by taking part in research

Can you draw yourself as an avatar? By taking part in this study you are a citizen scientist!

What is JU:MP?

JU:MP is aiming to get children active in Bradford! They are working with schools, the community, young people and people in charge to get children moving.

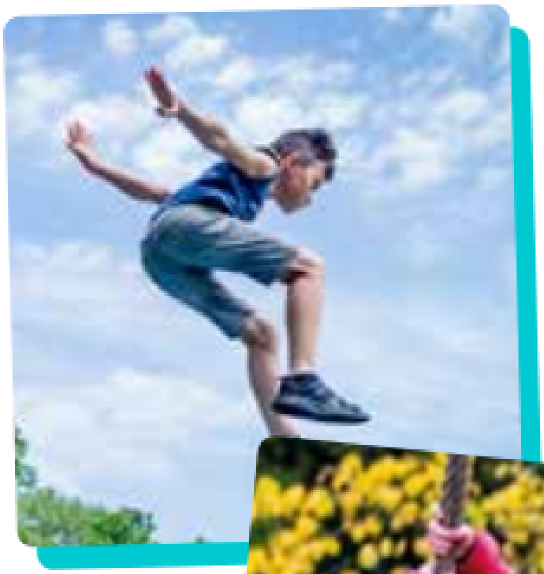

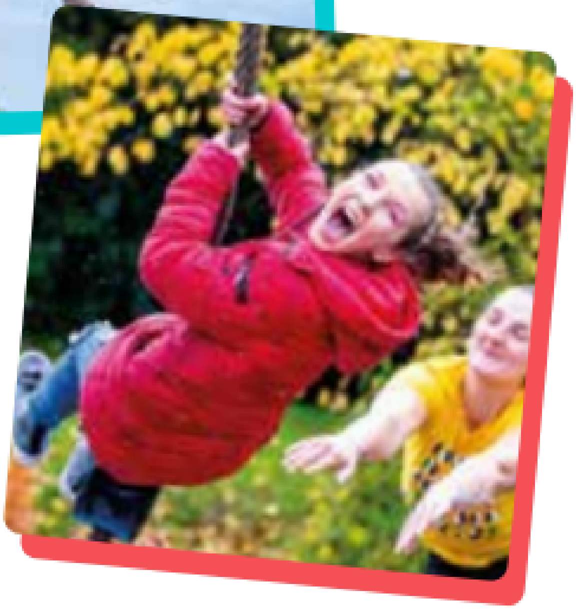

How do you like to move outside?

What is Bestlife?

Bestlife is an app designed by JU:MP and app developers to encourage children, their friends and family to be active. This could be by going on adventures to a local park or doing a group challenge like standing on one leg for as long as you can . If you do activities you earn points and can update your avatar.

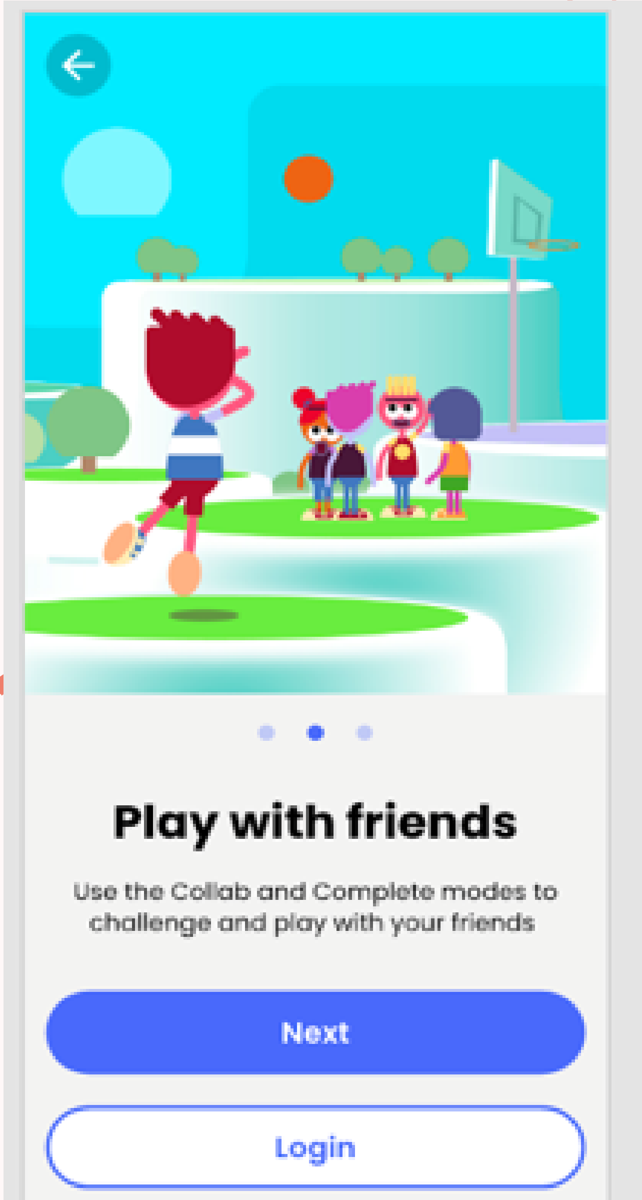

What exercise do you like to do at home?

What is this citizen science project all about?

Finding out what you and your family think about bestlife - good and bad!

Getting you involved in research through citizen science.

Listening and learning from you to improve how we carry out our research next time.

Do you want to take part? Why/Why not?

What am I going to do?

Download the bestlife app - check out the last page for help

Try bestlife over the next week as many times as you like

Think about how you have found using the app, what do you think about it?

Read and fill in this booklet

Ask your parents to sign the consent form so you can come to the next session and let us know how you have got on.

Quick Quiz

- **Is JU:MP**
  - a) a program to introduce Kangeroos to Yorkshire? ☐
  - b) aiming to get children in Bradford active ? ☐
  - c) a skipping workshop for people over 80 years old? ☐
- **Is citizen science**
  - Something you can only do in a lab with test tubes and fire? ☐
  - People like me creating new knowledge ☐
  - A job you can get paid to do ☐
- **Is bestlife**
  - An app to encourage children and families to be active ☐
  - An award for being awesome and having the best life? ☐
  - A new chocolate bar? ☐

First impressions of bestlife

These are some words we came up with when we thought about JU:MP

JU:MP
Bradford
Family
Bestlife
Child
Active
Outside
Parks

What words do you think of when you use bestlife?

What two things would you like to tell us about Bestlife?

1

2

Did you play bestlife with someone? - what did they think?

Gran

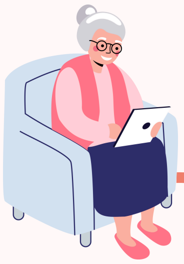

Gran liked coming on quests to the park

She didn't like the hair on the characters

How you are feeling

What did you think of being asked how you were feeling?

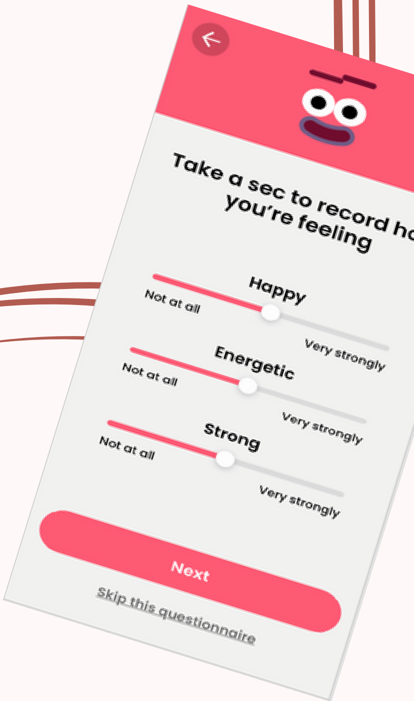

Would you have used the same words?

strong   happy   energetic

nervous   sad   angry

Any notes or questions you have

Young persons consent form

I have read my booklet and understood the information ☐

I understand that I don't have to do any of this, I don't have to fill this in or go to the citizen science session. If I decide after I don't want to be part of it I can let the researcher know within two weeks ☐

What I say will be anonymous, no one will know outside of the group who said what. (If I am in danger or need help, the researchers will tell the people at school who need to know) ☐

My quotes might be used in research but it wont have my name next to it ☐

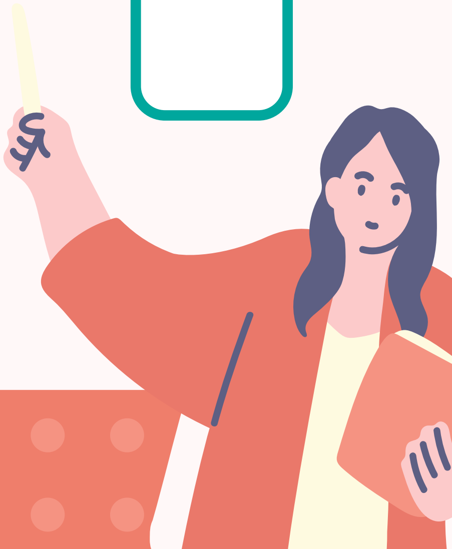

How to download the app

- Go to the app store on your phone
- Find the bestlife app
- It looks like this

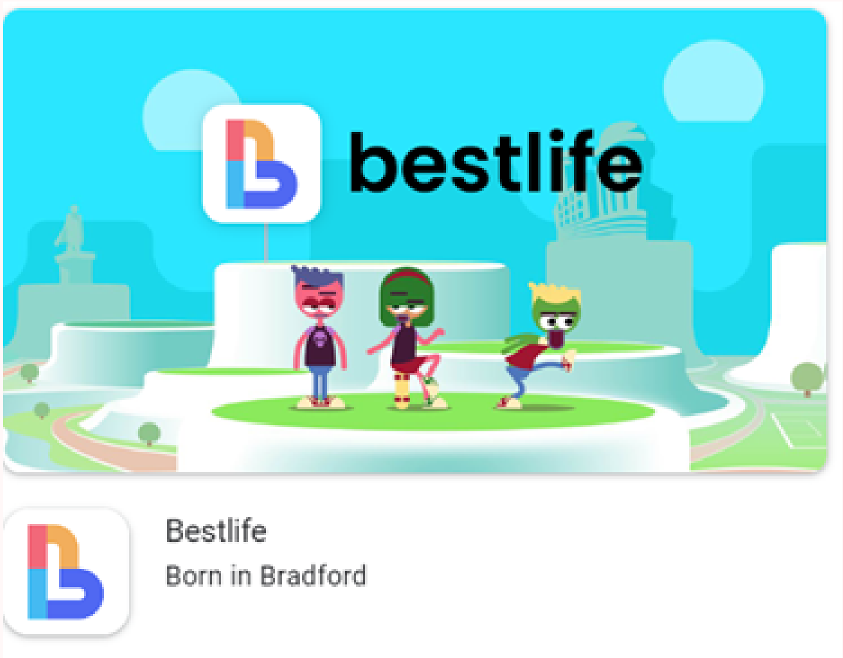

- Ask for parental approval to download if it is on your phone.
- You will need your parents to put in their email and a verification code will be sent to them to allow you to download the app.

Congratulations you are now ready to play bestlife.
