# Supplementary material for: Active Play in a Digital Age, Exploring Children’s (Aged 8-13 Years) Views of a Physical Activity App: Qualitative Formative Study
Source: JMIR Form Res. 2025 Nov 11;9:e76498. doi: 10.2196/76498 (PMC12614868; doi:10.2196/76498)
Supplement: Multimedia Appendix 3 [file formative-v9-e76498-s003.docx]

**Focus group guide** (approx. time 40 minutes including break)

**The questions may not be covered in this order depending on where the conversation is taken by the participants.**

| **Introductory Task** |
| --- |
| **Ask children to assent to taking part in the research.**  Ask children to feedback one/two of their key points from the booklets.  Prompts can be asked here if children need it, did you like the app, could you download it okay, do you want me to read what you have written? Prompts can also be asked around their answers e.g. why do you say that, are there any reasons for this etc. |
| **Feasibility and Usability** |
| How did you or your family find downloading the app?  Prompts   - Did you have any problems using it? - Could you access the internet okay? - Would anything have made this easier? - Did you find the app hard or easy to use? - Was there anything you didn’t understand in the app - Was there anything you wanted the app to do but it didn’t? |
| **Acceptability** |
| Children can use the page where they wrote down what people thought about the app in their booklets.  Prompts   - How do you feel about the app? Did you like it? Not like it? Did you use it often? Or hardly at all? Would you recommend it? What did other people think? - Did anyone do a solo challenge? What did you think about it? What did other people think? - Did you take part in a collaborative challenge what did you think abut it? Did you find someone to take part with you? What did other people think? - Did anyone go on a park quest? Tell me about it, who did you go with? Where did you go? What did you think about it? What did other people think? - What did you think of filling in your emotions? Did you like being able to see how you felt on the graph? |
| **Acceptability – impact** |
| - Did the app change your routine or what you did at all? - Did you download further apps or discover new areas etc? |
| **Conclusion** |
| - Is there anything else the children want to add or ask? |
