# Supplementary material for: Active Play in a Digital Age, Exploring Children’s (Aged 8-13 Years) Views of a Physical Activity App: Qualitative Formative Study
Source: JMIR Form Res. 2025 Nov 11;9:e76498. doi: 10.2196/76498 (PMC12614868; doi:10.2196/76498)
Supplement: Multimedia Appendix 4 [file formative-v9-e76498-s004.docx]

Code Book

| Theme | Code | Sub code | Key Quotes |
| --- | --- | --- | --- |
| **Feasibility** - the practicality of implementing the app within the target population's daily routines and local context [(Bowen et al., 2009)](https://www.zotero.org/google-docs/?LjHsdB). | Download | Downloading process | *"Yeah, because it’s only a small download, like 38 megabytes"* (Secondary 1)  Students would not have downloaded it without being prompted - *“I didn't really know about it”* (Secondary 2)  Where they downloaded it:  *“my dad's phone” “my phone”* (Year 5 Primary 2) |
|  |  | Parental consent | *"Having to have your parents is another thing that just will add annoyance to downloading it"* (Secondary 1)  *“I had to put my dad's password in, and he's always at work”* (Secondary 2)  *“At the time when I was downloading it, Mum and Dad were both at work so yeah”* (Secondary 1) |
|  | Social support (family and friends) |  | *“Mummy helps me with counting and doing the stopwatch”* (Year 4 Primary 1)  *“I played with mummy she was happy as she liked to see me being active and help me”* (Year 4 Primary 1)  *“Mummy liked playing star jump”* (Year 4 Primary 1)  *“Played it with my friend, she liked the skipping and being active”* (Year 6 Primary 1) |
|  | Time constraints | School | *"You’re kind of on, like especially in school, a tight timetable"* (Secondary 2).  *“Me and my family were really busy, so I don’t really go out walking often”* (Secondary 1) |
|  | Linking with existing activities |  | *“Junior Park Runs could be a good place to have the app done because it’s children doing physical activity”* (Secondary 1) |
|  | Outside environment | Suggesting areas nearby to include | *Several named parks mentioned and unnamed areas near their schools and houses e.g. recs and football pitches (Year 5 Primary 2) (Secondary 1)* |
|  |  | Use in the park | *“I was in the park, and I was doing like hanging that thing, then I was doing star jumps, and then I had to do a little run”* (Year 5 Primary 2)  *“miss I don't want to do it, I've got other things to be doing in the park" (Researcher: ‘Like what?’ ) “like playing football or going on the slides”* (Year 5 Primary 2) |
| **Acceptability** - How children react to the app, do they think it is appropriate, satisfactory and attractive [(Bowen et al., 2009)](https://www.zotero.org/google-docs/?ZSLM03) | General |  | *“It encourages us to be active”* (Year 5 Primary 2)  *“Decent activities”* (Year 5 Primary2 )  *“It's very active, so I like it” (*Year 5 Primary 2)  *“I loved how it was so active”* (Year 5 Primary 2)  *“I thought it was quite, quite and entertaining game”* (Year 6 Primary 2)  *“It’s like, fun to get points for doing activities like jumping jacks”* (Year 5 Primary 2)  *“It has a variety of activities” (Year 5 Primary 2)* |
|  | Reward System | Immediate gratification | (child jumping up and down exclaiming) *"We got 20 points!"* (Year 6 Primary 2)  *“Earning points and having to work for them”* (Year 4 Primary 1)  *“I like it because you get points”* (Year 4 Primary 1) |
|  |  | Proportional rewards | *"10 or less, you should get 10 points; 10 to 20—you should get 20. And then if it’s 30 or more, then you can have 50"* (Secondary 1).  *“If someone worked harder, they should get more points”* (Year 6 Primary 1)  *“I don't like how it gives you less points for some of the activities”* (Year 5 Primary 1)  (Researcher: Would you go to a park and scan a code to get some points) *“No thanks”* (Researcher: Okay, why did you say no?) *“I want to work for them”* (Year 5 Primary 2) |
|  | Customisation | Avatar design and customisation | *“I think the outfits are good”* (Year 6 Primary 2)  *"I liked that you could change clothes, but we need more colours and fun stuff like dresses or branded items like Nike."* (Year 6 Primary 1).  Children wanted more store items, including: *“Skipping ropes, hula hoops, footballs, basketballs”* (Year 6 Primary 1)  *“Footballs”, “headphones” “golden guitar” “emerald chain” “glasses” “pets”* (Year 5 Primary 2)  *“football jerseys”, “pets”, “hoodies”, “branded clothing”* (Year 4 Primary 1)  *“Emo avatar and clothes”* (Year 5 Primary 2)  *“cats”, “dogs”, “birds”, “snakes” “pandas”* (Year 6 Primary 2)  *“Unicorns”, “butterflies”, “grasshoppers”, “tigers” “cats” “leopards”* (Year 5 Primary 1)  *“A way to design your own avatar”* (Year 5 Primary 2)  *“More facial expressions”* (Year 6 Primary 2)  Wanted more avatar customisation options, including *“I am going to draw a Hijab”* (Year 4 Primary 1)  *“Shoes are weird”* (Year 6 Primary 2)  *“A hijab” ‘‘abaya” “more hijabs and jilbabs”* (Year 5 Primary 2)  *“More skin colours”* (Year 5 Primary 2) |
|  |  | Themes and backgrounds | *“Maybe a light and dark mode or something like that”* (Secondary 1)  *“Change the background”* (Year 5 Primary 2)  *“Different backgrounds”* (Year 5 Primary 2)  *“I like the background”* (Year 5 Primary 2)  *“Do you know when it‘s Halloween or Christmas - there should be special gear for that”* (Year 6 Primary 2)  *“Christmas theme or summer outfits”* (Year 5 Primary 2) |
|  | Updates |  | *“Every so often it just needs to have an update to keep it being exciting”* (Secondary 2) |
|  | Activity types | Non sport related | *“Cooking and cleaning” and “Singing” - suggested activities (Year 6 Primary 1)*  *“It would persuade you to do it. I’m not going to lie I’m quite lazy when my days go do your chores [name]. So it could be a way to persuade. Competitiveness with yourself.” (Year 5 Primary 2)*  *“5-minute timer to do a chore and you get points” (Year 5 Primary 2)*  *“Music”, “Being mindful” (Year 5 Primary 2)* |
|  |  | Alternative sports related activities | *“WWE” - suggested activities (Year 6 Primary 1)*  *“If dance was added, I would have used the app a little bit more” (Secondary 1)*  *“20 minutes playing sports” (Year 5 Primary 2)*  *“Dance” (Year 5 Primary 2)*  *“Racing”, “gymnastics”, “dancing” “jogging on the spot” “football” “traffic lights” “basketball” “dance to the beat”, “cricket” (Year 5 Primary 2)* |
|  |  | Tutorials | *"I don’t know how to do push-ups, but I tried. Maybe a tutorial or video would help."* (Year 5 Primary 2 Year 5). |
|  | Cultural relevance |  | *"That’s one thing I’m like, where are the hijabs? This is for people in Bradford"* (Secondary 2).  “*There was no hijabs, I didn't see any”* (Secondary 1)  Wanted more avatar customisation options, including *“I am going to draw a Hijab”* (Year 4 Primary 1)  *“A hijab” ‘‘abaya” “more hijabs and jilbabs”* (Year 5 Primary 2) |
|  | Feelings about activity |  | *"It just like reminds you how you feel when you do like different types of things,"* (Secondary 1)  *“Tiring”* and *“exhausting”* were used to describe the activities (Year 4 Primary 1) |
|  |  | Sound effects | *“When you press a button it makes a noise”* (Year 5 Primary 2) |
|  |  | Avatar animation | *“If there was an animation while running, it would be more interesting” (Secondary 1)* |
|  |  | Feeling descriptions | *“Over the moon” “overjoyed” “furious” and “ecstatic” were all mentioned as different emotion words* (Year 6 Primary 1)  *“Strong like a bodybuilder” - confusion over word definition* (Year 6 Primary 1)  *“Crazy’ full of life” “as crazy as a gorilla”* (Year 5 Primary 2)  *“I would like to be asked because i know people care” (Year 5 Primary 2)*  *“I would be happy to do it”* (Year 5 Primary 2)  *“I’d use the same words” (*Year 6 Primary 2)  *“Entertained”* (Year 6 Primary 2) |
|  | Socialisation and Collaboration | Competition | *"It’d be fun to see how I’m doing compared to my friends"* (Year 6 Primary 2)  *"If you do it at school, there’s more people being competitive, like in a class,"* (Secondary 1).  *“I feel like there could have been a race between people so we could have done more”* (Year 6 Primary 1)  Interest in school-wide competition - *“Maybe in like, if it becomes a larger scale project, maybe like forms at school”* (Secondary 1)  *“We got 40 points! I like that we can compete with others to see who’s better”* (Year 6 Primary School 1).  “I liked seeing how much I did and comparing it with others” (Year 6 Primary 1) |
| **Usability** - Usability focuses on the design aspects of the app that contribute to a positive user experience [(Wu et al., 2017)](https://www.zotero.org/google-docs/?rlNY16). | Collaboration/sharing |  | *"It’s more fun to do the activities with someone, like when we shared the tablet."* (Year 5 Primary 2). |
|  | Functionality | Linking to other devices | *"My phone with Samsung Health app automatically tracks my steps. If that was implemented, it would also see the steps"* (Secondary 1).  *“I want to track my heart rate”* (Year 6 Primary1)  *“I want to check my heartbeat”* (Year 4 Primary 1)  *“You know the step activities, does the app track your steps?”* (Year 4 Primary 1)  Suggestion to connect with step-tracking devices - *“I do have a watch which I used to count my steps and my phone automatically counts my steps”* (Secondary 2) |
|  |  | Downloading on other devices | *“In BestLife I couldn’t do it on my laptop”* (Year 6 Primary 1)  *“I want to do it on my Switch”* *(Year 6 Primary 1)* |
|  |  | Prompts | *“Daily reward to make people want to play it more”* (Secondary 2)  *Emotional tracking reminders suggested - “If a notification popped up? Yeah, It’d make you remember to go on the app… like a lot of the time I won’t remember to go on any apps”*(Secondary 2)  *“Maybe if you go in the app for way too long, there's a message that says like do you want to take a break now?” (Year 6 Primary 2)* |
|  | Clarity of features |  | *“When I went on, I didn’t know what ‘solo quest’ meant"* (Secondary 2)  Confusion over an avatar's feature: *“Why is the avatar doing star jumps?”* (Year 4 Primary 1)  *“Usually when you download something, it pops up with guidance, but there wasn’t that”* (Secondary 2)  *“When I went on to it, it just starts at start your solo quest and I didn’t know what that meant”* (Secondary 2)  *“I find it really annoying when it forces a full tutorial”* (Secondary 1) |
|  | App navigation |  | *"It’s cool to see all the things I’ve done,"* (Secondary 1) - talking about the activity dashboard.  *"It was fun, but it was annoying when I couldn’t figure out how to go to the next task. The arrows were hard to see"* (Year 4 Primary 1).  *“Those graphs probably wouldn’t be that easy to find”* (Secondary 1) (talking about dashboards.) |
|  |  | Count down | *“Maybe like something to do, so say if you did like a five-minute activity and you just stood there watching the clock come down, then it's not gonna be like, very engaging, is it? Because there's, if there's like an animation or something, especially for younger kids…” (Secondary 1)*  *“Something that they could watch and something that they could do whilst, whilst they were like, on their five, on like a five-minute run or something. Because otherwise they are just stood there running, watching their clock countdown.” (Secondary 1)* |
| **Behaviour Change** | Motivation and Engagement - This theme encompasses the internal and external processes that initiate and sustain behaviour change. It includes factors that make activities appealing and encourage continued participation. | External Rewards | *“I like the store.”* (Year 6 Primary 2)  *“Referring to a better way to use points, ‘you kind of unlock new exercises’.”* (Year 6 Primary 2)  *“Getting rewards every day made me want to keep play”* |
|  |  | Intrinsic Rewards | *“I enjoyed doing the push-ups because it helps my body.”* (Year 4 Primary 1)  *“Child excitedly chants Name I’m doing a push-up name I’m doing a push-up.”* (Year 5 Primary 2)  *“Riding my bike is fun, I don’t need points for that.”* (Secondary 1) |
|  |  | Social Influence | *“It's more fun to do the activities with someone, like when we shared the tablet.” (Secondary 2)*  *“I'd probably end up doing it if my mum nagged at me hard enough” (Secondary 1)* |
|  |  | Feedback and Monitoring | *“It's kind of like, nice, because you can tell your real score.”* (Year 6 Primary 2)  Emotional tracking - *“Once a day would be better”* (Secondary 2)  *“Getting a cheer when you finish a task feels good.”* (Secondary 1) |
|  | Goal-Setting and Achievement - This theme involves establishing clear, measurable objectives and the processes involved in working towards and achieving these targets. | Short-term Goals | *“Let’s see if we got more than 20.”* (Primary 1 Year 4)  *“We set a goal to do 1000 steps a day.”* (Secondary 1) |
|  |  | Long-term Goals | *“Trying to reach the next level made me keep going.”* (Secondary 2)  *“It’s cool to see all the things I’ve done.”* (Secondary 2) |
|  |  | Collaborative Achievement | *“You can do a collab with them too, and it was really fun.”* (Year 4 Primary 1)  *“I like collab because you can invite friends over.”* (Year 5 Primary 2) |
|  | Effort and Capability- This theme addresses the individual's capacity to perform a behaviour and the perceived relationship between the effort invested and the rewards received. | Effort and Reward | *“If someone worked harder, they should get more points.”* (Year 6 Primary 1)  *“I think you should get more points the longer it goes.”* (Secondary 2)  *“More points for doing jumping jacks than walking.”* (Secondary 2) |
|  |  | Self-Assessing Capabilities | *“I actually don’t know how to do push-ups.” (Year 4 Primary 1)*  *“I know I’m getting better because I can do more steps now.” (Secondary 1)* |
|  |  | Building Skills   \|  \| \| --- \|  \|  \| \| --- \| | *“I don’t know how to do push-ups, but I tried. Maybe a tutorial or video would help.”* (Year 5 Primary 2) |
|  | Social Interaction and Support - This theme highlights the role of interpersonal relationships and social networks in facilitating behaviour change. | Peer Influence and Support | *“It’s good because you can collab with your friends.” (Secondary 1)*  *“Doing it with my brother made it less boring.” (Secondary 2)* |
|  |  | Family Involvement | *“Mummy helps me with counting and doing the stopwatch” (Year 4 Primary 1)*  *“My dad said it’s good that I’m exercising more.” (Secondary 2)* |
|  |  | Social Comparison and Competition | *“If you do it at school, there’s more people being competitive, like in a class.” (Secondary 2)* |
