# Supplementary material for: Active Play in a Digital Age, Exploring Children’s (Aged 8-13 Years) Views of a Physical Activity App: Qualitative Formative Study
Source: JMIR Form Res. 2025 Nov 11;9:e76498. doi: 10.2196/76498 (PMC12614868; doi:10.2196/76498)
Supplement: Checklist 1 [file formative-v9-e76498-s005.pdf]

Consolidated criteria for reporting qualitative studies (COREQ): 32-item checklist. Completed for the Bestlife formative qualitative study.

No. Item	Guide questions/description	Reported on Page #
Domain 1: Research team and reflexivity		
Personal Characteristics		
1. Inter viewer/facilitator	Which author/s conducted the inter view or focus group?	Page 7
2. Credentials	What were the researcher's credentials? E.g. PhD, MD	Page 7
3. Occupation	What was their occupation at the time of the study?	Page 7
4. Gender	Was the researcher male or female?	Page 7
5. Experience and training	What experience or training did the researcher have?	Page 7
Relationship with participants		
6. Relationship established	Was a relationship established prior to study commencement?	Page 7
7. Participant knowledge of the interviewer	What did the participants know about the researcher? e.g. personal goals, reasons for doing the research	Page 7
8. Interviewer characteristics	What characteristics were reported about the inter viewer/facilitator? e.g. Bias, assumptions, reasons and interests in the research topic	Page 7

Domain 2: study design		
Theoretical framework		
9. Methodological orientation and Theory	What methodological orientation was stated to underpin the study? e.g. grounded theory, discourse analysis, ethnography, phenomenology, content analysis	Page 8
Participant selection		
10. Sampling	How were participants selected? e.g. purposive, convenience, consecutive, snowball	Page 6
11. Method of approach	How were participants approached? e.g. face-to-face, telephone, mail, email	Page 6
12. Sample size	How many participants were in the study?	Page 7
13. Non-participation	How many people refused to participate or dropped out? Reasons?	Page 7
Setting		
14. Setting of data collection	Where was the data collected? e.g. home, clinic, workplace	Page 7
15. Presence of non-participants	Was anyone else present besides the participants and researchers?	Page 7
16. Description of sample	What are the important characteristics of the sample? e.g. demographic data, date	Page 6
Data collection		
17. Interview guide	Were questions, prompts, guides provided by the authors? Was it pilot tested?	Page 7
18. Repeat interviews	Were repeat inter views carried out? If yes, how many?	No
19. Audio/visual recording	Did the research use audio or visual recording to collect the data?	Page 7
20. Field notes	Were field notes made during and/or after the interview or focus group?	No
21. Duration	What was the duration of the inter views or focus group?	Page 7
22. Data saturation	Was data saturation discussed?	No
23. Transcripts returned	Were transcripts returned to participants for comment and/or correction?	No
Domain 3: analysis and findings		
Data analysis		Page 8

24. Number of data coders	How many data coders coded the data?	Page 8-9
25. Description of the coding tree	Did authors provide a description of the coding tree?	Page 8
26. Derivation of themes	Were themes identified in advance or derived from the data?	Page 8
27. Software	What software, if applicable, was used to manage the data?	Page 8
28. Participant checking	Did participants provide feedback on the findings?	No
Reporting		
29. Quotations presented	Were participant quotations presented to illustrate the themes/findings? Was each quotation identified? e.g. participant number	Page 9-13
30. Data and findings consistent	Was there consistency between the data presented and the findings?	Page 12-13
31. Clarity of major themes	Were major themes clearly presented in the findings?	Page 9-13
32. Clarity of minor themes	Is there a description of diverse cases or discussion of minor themes?	Page 12-13
